# Development of an image-based screening system for inhibitors of the plastidial MEP pathway and of protein geranylgeranylation

**DOI:** 10.12688/f1000research.5923.2

**Published:** 2015-08-12

**Authors:** Michael Hartmann, Elisabet Gas-Pascual, Andrea Hemmerlin, Michel Rohmer, Thomas J. Bach

**Affiliations:** 1 Département “Réseaux Métaboliques, Institut de Biologie Moléculaire des Plantes, CNRS UPR 2357, Université de Strasbourg, 28 rue Goethe, F-67083 Strasbourg, France; 2 UMR 7177 CNRS/Université de Strasbourg, Institut Le Bel, 4 rue Blaise Pascal, F-67070 Strasbourg, France; 3 Current address: Department Biologie, Institut für Molekulare Ökophysiologie der Pflanzen, Universität Düsseldorf, Universitätsstr. 1, D-40225, Düsseldorf, Germany; 4 Current address: Horticulture and Crop Science, Ohio State University, 208 Williams Hall, 1680 Madison Avenue, Wooster, OH, 44691, USA

**Keywords:** protein geranylgeranylation, tobacco BY-2, protein geranylgeranyl transferase type 1, cytosolic isoprenylation, fluorescence microscopy

## Abstract

In a preceding study we have recently established an *in vivo* visualization system for the geranylgeranylation of proteins in a stably transformed tobacco BY-2 cell line, which involves expressing a dexamethasone-inducible GFP fused to the prenylable, carboxy-terminal basic domain of the rice calmodulin CaM61, which naturally bears a CaaL geranylgeranylation motif (GFP-BD-CVIL). By using pathway-specific inhibitors it was there demonstrated that inhibition of the methylerythritol phosphate (MEP) pathway with oxoclomazone and fosmidomycin, as well as inhibition of protein geranylgeranyl transferase type 1 (PGGT-1), shifted the localization of the GFP-BD-CVIL protein from the membrane to the nucleus. In contrast, the inhibition of the mevalonate (MVA) pathway with mevinolin did not affect this localization. Furthermore, in this initial study complementation assays with pathway-specific intermediates confirmed that the precursors for the cytosolic isoprenylation of this fusion protein are predominantly provided by the MEP pathway. In order to optimize this visualization system from a more qualitative assay to a statistically trustable medium or a high-throughput screening system, we established now new conditions that permit culture and analysis in 96-well microtiter plates, followed by fluorescence microscopy. For further refinement, the existing GFP-BD-CVIL cell line was transformed with an estradiol-inducible vector driving the expression of a RFP protein, C-terminally fused to a nuclear localization signal (NLS-RFP). We are thus able to quantify the total number of viable cells versus the number of inhibited cells after various treatments. This approach also includes a semi-automatic counting system, based on the freely available image processing software. As a result, the time of image analysis as well as the risk of user-generated bias is reduced to a minimum. Moreover, there is no cross-induction of gene expression by dexamethasone and estradiol, which is an important prerequisite for this test system.

## Introduction

In eukaryotic cells, certain proteins ( *i.e*., members of the Ras superfamily of GTPases in vertebrates) are modified by a series of post-translational modifications, leading to the creation of a lipidated, hydrophobic domain at the carboxyl terminus of the protein. This post-translational processing, also referred to as protein isoprenylation, mediates protein-protein interactions, increases the affinity of the prenylated proteins to cellular membranes and is therefore important for the targeting and function of such covalently modified proteins. Protein isoprenylation depends on the presence of a carboxy-terminal tetrapeptide in target proteins, the CaaX motif (‘C’ refers to cysteine, ‘a’ denotes an aliphatic amino acid and ‘X’ represents a specific amino acid) ^1– 4^.

This C-terminal CaaX motif is recognized by cytosolic CaaX protein isoprenyl transferases, which either attach a 15-carbon farnesyl unit (from farnesyl diphosphate, FPP), a reaction catalyzed by a protein farnesyl transferase (PFT), or a 20-carbon geranylgeranyl unit (from geranylgeranyl diphosphate, GGPP), a reaction catalyzed by protein geranylgeranyl transferase (PGGT1) to the cysteine of the CaaX motif *via* a thioether bond. The specificity of the reaction is mainly defined by the C-terminal “X”. As a general rule, proteins are geranylgeranylated when the “X” is a leucine residue, whereas any other amino acid - most probably a methionine, serine, alanine, glutamine or cysteine - will lead to the covalent attachment of a farnesyl residue ^5^. A third mechanism is known for the members of the Rab family of small GTPases, which are isoprenylated at two different C-terminal cysteine residues by Rab geranylgeranyl transferase (also referred to as PGGT2) ^6– 8^. Both PFT and PGGT1 are heterodimeric enzymes that share a common α-subunit whereas their respective β-subunit is encoded by different genes ^3, 4, 6, 9^.

Following prenylation in the cytosol, the newly lipidated protein is targeted to the endoplasmic reticulum (ER), where it usually undergoes two subsequent processing reactions; first, the C-terminal amino acid is removed by a specific endoprotease, known as RCE1 (RAS converting enzyme 1); second, the carboxyl group of the now exposed, lipidated cysteine residue is carboxyl-methylated by the enzyme isoprenylcysteine carboxyl methyltransferase (ICMT). In contrast to the prenylation reaction and the proteolytic removal of the -AAX tripeptide, this last step in the maturation of prenylated proteins can be reversed by certain methylesterase enzymes (ICME), which have been identified in animals and plants ^10– 14^. The proteolytic cleavage of the last three amino acids and the carboxyl-methylation are commonly referred to as “CaaX processing” or post-prenylation reactions ^15^.

In addition to these post-translational modifications, certain proteins, such as NRAS, HRAS and KRAS4A in vertebrates or members of the Rop (Rho) family in plants can be palmitoylated as well or *S*-acylated, preceding their transfer to their cellular destination - most likely the plasma membrane ^16– 19^. Other proteins, such as KRAS4B do not require a second lipidic modification, but possess a polybasic, lysine-rich sequence in proximity to the C-terminal CaaX motif instead ^18^.

Prenylated proteins have been particularly well studied in vertebrates because of their implication in oncogenesis, as mutational constitutive activation of RAS GTPases is responsible for up to 20% of human cancers ^20– 22^. In comparison, less is known about prenylated proteins in plants, even though many studies suggest that these proteins play important roles in cellular processes similar to their yeast and mammalian counterparts, such as growth regulation, signal transduction, cell cycle regulation and membrane trafficking ^4, 6, 9, 23– 27^.

Following earlier observations with a specific calmodulin from *Petunia* (CaM63 ^28, 29^, we have established a system for the visualization of protein geranylgeranylation, based on a dexamethasone-inducible GFP fusion protein, N-terminally fused to the C-terminal extension of rice calmodulin CaM61, which bears a basic domain (BD) plus a CVIL geranylgeranylation motif using stably transformed tobacco BY-2 cells ^30– 32^. After the covalent modification of the GFP-BD-CVIL protein (or of its His _6_-tagged derivative H _6_-GFP-BD-CVIL) it becomes predominantly localized to the plasma membrane. The non-prenylatable control protein GFP-BD-C/S (GFP- BD-SVIL), in which the cysteinyl residue of the CVIL-motif had been replaced by a serinyl residue (thus removing the thiol group necessary for the covalent attachment of the prenyl moiety), nearly completely mislocated to the nucleus and in particular to the nucleoli of the cells. During the course of such studies it was revealed that the inhibition of the cytosolic MVA pathway by mevinolin had no effect on the geranylgeranylation-dependent insertion of the fusion protein into the plasma membrane ^30, 31^. This is in contrast to inhibition of the MEP pathway, for instance by oxoclomazone, which was previously recognized as an inhibitor of DXP synthase ^33, 34^ and with fosmidomycin, acting on MEP synthase ^35, 36^, the second enzyme in this pathway. We immediately thought of developing this system further into a versatile screening system capable of demonstrating the *in vivo* efficiency of MEP pathway (and protein geranylgeranylation) inhibitors ^30^, though this required the miniaturization of the system to allow the use of fluorescence and confocal microscopy.

Our contribution here is in focusing on the key steps that are necessary for the development of a screening pipeline ^37, 38^ and to demonstrate the feasibility of establishing a medium to high throughput compound screen on the basis of our *in vivo* protein isoprenylation assay ^30, 32^.

### General considerations as to medium and high throughput screening methods based on fluorescence microscopy

Over the last decade, technological advances have dramatically changed the importance of image-based assays for modern cell biology. In the past, classical, non-microscopic approaches have been systematically used to investigate protein functions and interactions or to screen small-compound libraries in high-throughput. Thanks to the knowledge and tools developed during the evolution of various technologies in the last few years, including proteomics and genetics approaches ^39– 42^ and DNA microarrays ^43– 45^ or RNA interference experiments ^46– 48^, fluorescence microscopy has become a powerful method to study protein functions and interactions in the living cell ^45, 49^. This was in particular accomplished due to the availability of a great variety of fluorescent proteins and fluorophores ^50– 54^, which can be used to tag a protein of interest and to reveal information about its localization, its interaction with other cellular components and proteins or even to visualize biochemical reactions, *e.g.* the effect of a given treatment at a cellular level. This may permit conclusions on the physiological relevance of the protein, in contrast to classical, biochemical assays, where an isolated protein is tested in an artificial environment. In this way, data acquired by fluorescence microscopy can help to complement the above mentioned genetic and proteomic approaches.

Because of the availability of automated microscopes and more powerful image analysis software, multiple features of a cell can be measured, analyzed and compared, even over a certain period of time.

Previously, researchers were often forced to inspect their acquired microscopic images by eye, which is a time-consuming and subjective task, even for an experienced user ^55– 57^. Nowadays, modern, automated image acquisition platforms provide highly quantitative data and allow image-based screens of several thousand compounds a day, depending on the experimental set-up. In addition, fluorescence microscopy also allows to follow reactions at different scales. For instance, changes at the subcellular level can be observed using high resolution ^58, 59^, whereas a population of cells can be monitored using low resolution ^60, 61^, thereby providing a large set of data for every single image. This combination of high throughput-screening (HTS) methods and automated image acquisition and analysis has therefore been dubbed high-content-screening ^37, 38, 62– 68^.

Nevertheless, image-based chemical screening still remains a field in development, with the majority of users belonging to the pharmaceutical industry ^69^. As the technology is being pushed by those companies, the variety of HCS systems available to the scientific community and the number of publications generated with them has considerably increased over the past few years ^69– 73^. However not every academic research unit having developed a biological assay with the potential for high-throughput/content screening will be able to use modern imaging platforms, as all the hardware and software components of a complete screening pipeline (not counting the time for development and validation) represent a significant financial investment ^68^.

Another trend in high-content screening microscopy involves a multidimensional image read-out. This approach is also known as multiplexing ^74, 75^. Technically, it consists of using multi-color fluorescence microscopy and several distinct fluorescent markers in the same cell system. Of course, the spectral properties of the used fluorescent dyes and fluorophores as well as the detection gain and the computing ability of the microscopy platform are limiting factors, although this can be partially resolved by linear unmixing of fluorescent signals, “ *a method allowing the reliable separation of even strongly overlapping fluorescence signals*...” ^76, 77^. Multiplexing is particularly interesting if more than one phenotype needs to be analyzed. As a side effect, changes in the morphology of the cells can also be monitored, which can reveal a potential toxicity event during the screening experiment. For example, multiple fluorophores, staining the nucleus, cytoplasm, microtubules, Golgi or endoplasmic reticulum could be detected in parallel, revealing additional information about cellular changes, as part of a small compound screen ^78, 79^. As a result, a pre-selection of drug-candidates can be performed at an early stage in the whole screening pipeline. This aspect can be quite important, considering that up to 30% of potential drug candidates are rejected because of toxicity issues ^80^. Likewise, efficiency can be increased and the costs significantly reduced ^64, 80^.

However, one of the most critical steps remains the analysis of images, especially in purely cell-based assays. Although there is a variety of commercial software available for numerous purposes, many applications - especially those with complex cellular phenotypes - require custom-made solutions.

By successfully testing the robustness and selectivity of the test system based on transformed BY-2 cells, and also the efficiency of some novel drug candidates *in vivo* ^32^, we had now established this bioassay as a qualitative and quantitative approach for the identification and evaluation of MEP pathway and eventually of protein geranylgeranylation inhibitors. However, all analyses performed so far with this bioassay were reliant on the performance of a single microscope user, observing biological processes and counting individual cells through the ocular of a microscope. Therefore, further developments were required, in particular to reduce the involuntary bias of results by the user and to increase the speed and reproducibility of the application.

Some important aspects can escape the human eye during a visual observation, and sometimes it is difficult to draw conclusions of biological significance by analysis of single cells. Therefore, it was important to check the reproducibility of the results with a random collection of cells. Furthermore, the quality of the acquired digital images had to be high enough for an automatic identification and measurement of biological features, such as the nuclear fluorescence observed in inhibitor-treated BY-2 cells. The use of BY-2 cells as a suitable model to investigate the effects of various treatments on the whole cell level has various advantages, including the efficient uptake of exogenous compounds and the short reproduction time. However, an important problem was the heterogeneity of fluorescence of liquid suspensions derived from the first-generation calli. We successfully solved this problem and this was an important prerequisite for the further development of a statistically significant screening system ^32^.

Therefore, a major emphasis of this study was to further improve the initial test system and to evaluate its potential for miniaturization, automatization and, if possible, high-throughput applications. However, the results obtained after treatment of the H _6_-GFP-BD-CVIL lines with squalestatin demonstrated that the effects on the morphology and localization of the fusion protein could be more complex than initially expected (due to partial delocalization, roundish cell shape and “all-or-nothing” – phenotype ^32^).

## Methods & results

### Development of an image-based chemical screening system for inhibitors of the plastidial MEP pathway

**Figure 1.**
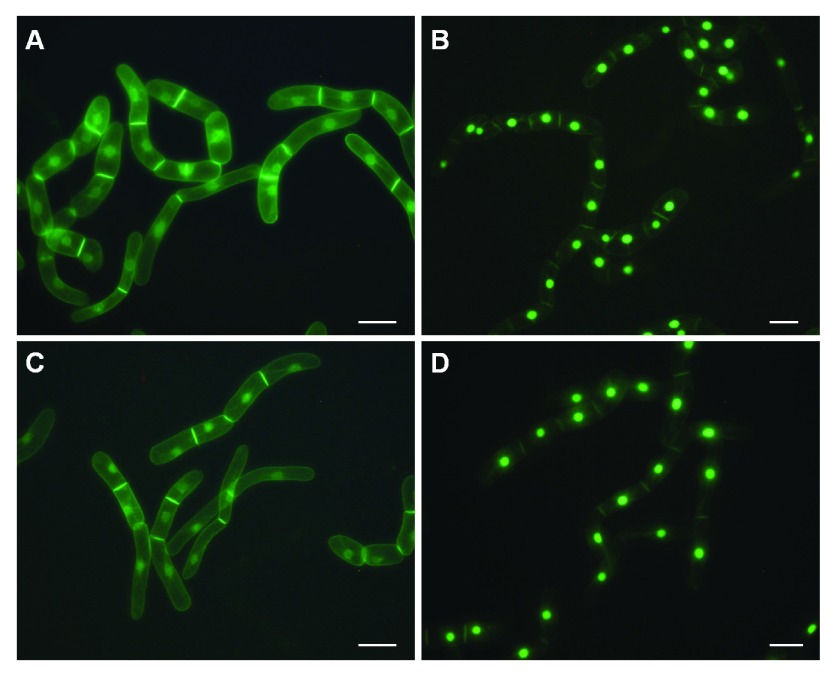
Reproducibility of results on a population of treated BY-2 cells. Low magnification fluorescence images of large numbers of cloned BY-2 cells expressing H _6_-GFP-DB-CVIL (cf. Gerber *et al.*, 2009). Images were taken with a Nikon E800 microscope equipped with a DXM11200 CCD color camera (specifications: 20 x 0.45 objective; filters: Ex460–500, DM505, EM510–560). **A**: Untreated cells; **B**– **D**: cells treated with 30 µM oxoclomazone (OC, **B**), 5 µM mevinolin (MEV, **C**) and 30 µM OC plus 5 µM MEV ( **D**). The white bar represents 50 µm.

In order to demonstrate the feasibility and reproducibility of such an image-based approach, BY-2 cells expressing the H _6_-GFP-BD-CVIL fusion protein was treated with the MEP- or MVA pathway inhibitors, oxoclomazone (OC, 30 µM, synthesized by Dr. Eilers, Monsanto, St Louis, MO) or mevinolin (MEV, 5 µM, made available by MSD, Rahway NJ) respectively, as well as with a combination of both inhibitors according to standard protocols ^30^. Images were acquired at low magnification using a Nikon E800 microscope. Low magnification fluorescence microscopy uses a resolution of 500 µm to 1 mm and is used to monitor phenotypic effects on entire cell populations. Medium-magnification fluorescence microscopy by definition is applied for subpopulation analysis at a resolution between 10–50 µm, whereas high-magnification fluorescence microscopy focuses on intracellular events and uses resolution of 1 µm or lower ^56^. The phenotypes of the untreated control and the MEV-treated cells were more or less identical, with the majority of fluorescence at the cell periphery, especially at the boundary between cells in the files. In addition, GFP fluorescence was also clearly visible at the peri-nuclear membrane, whereas the GFP fluorescence in OC-treated cells was mostly localized in the nucleus. The combination of both inhibitors gave about the same or even more unequivocal results than cells treated with OC alone ( Figure 1, cf. 30).

### Image acquisition and analysis

For image analysis, we first used ImageJ, version 1.41 – 1.43, a publicly available Java-based image-processing program, which was inspired by NIH Image for the Macintosh ^81^. The MBF ImageJ bundle used in this study provided an excellent selection of the most important plug-ins for microscopy users due to a well-illustrated online manual. At this point it should be noted that several other free image processing software programs are available nowadays that allow to customize the analysis of microscopic images and to generate tailor-made solutions for the identification and evaluation of complex phenotypes in experimental datasets ^82– 84^.

One of the major aspects when working in fluorescence microscopy is the level of saturation of the images. For instance, within an optimal scenario, all images taken in the green channel – displaying the localization of the geranylgeranylatable GFP fusion protein - should be acquired with a minimum of saturation to reduce signals corresponding to unspecific background noise or non-specific fluorescence associated with cellular structures. For the subsequent image analysis, it is quite important to differentiate any object from its surrounding background. This process is referred to as “segmentation”. Most of the software programs operating in the microscopes and used for image acquisition, such as the LSM image browser or the AxioVision software, offer the option to control the saturation levels of the images during or after the acquisition process.

The general principle of the particle analysis with ImageJ or similar software packages can be explained by using an image of 30 µM OC-treated cells (acquired with the E800 epifluorescence microscope from Nikon), where the delocalization of the GFP-BD-CVIL protein is clearly visible.

The overall process can be divided into the following parts:

i “thresholding”
ii “watershed separation”
iii “particle analysis”.

**Figure 2.**
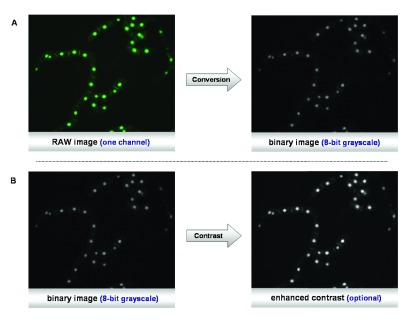
First steps of the segmentation process. **A**) Conversion of the RAW color image to a binary image file. **B**) Enhancement of the contrast.

**Figure 3.**
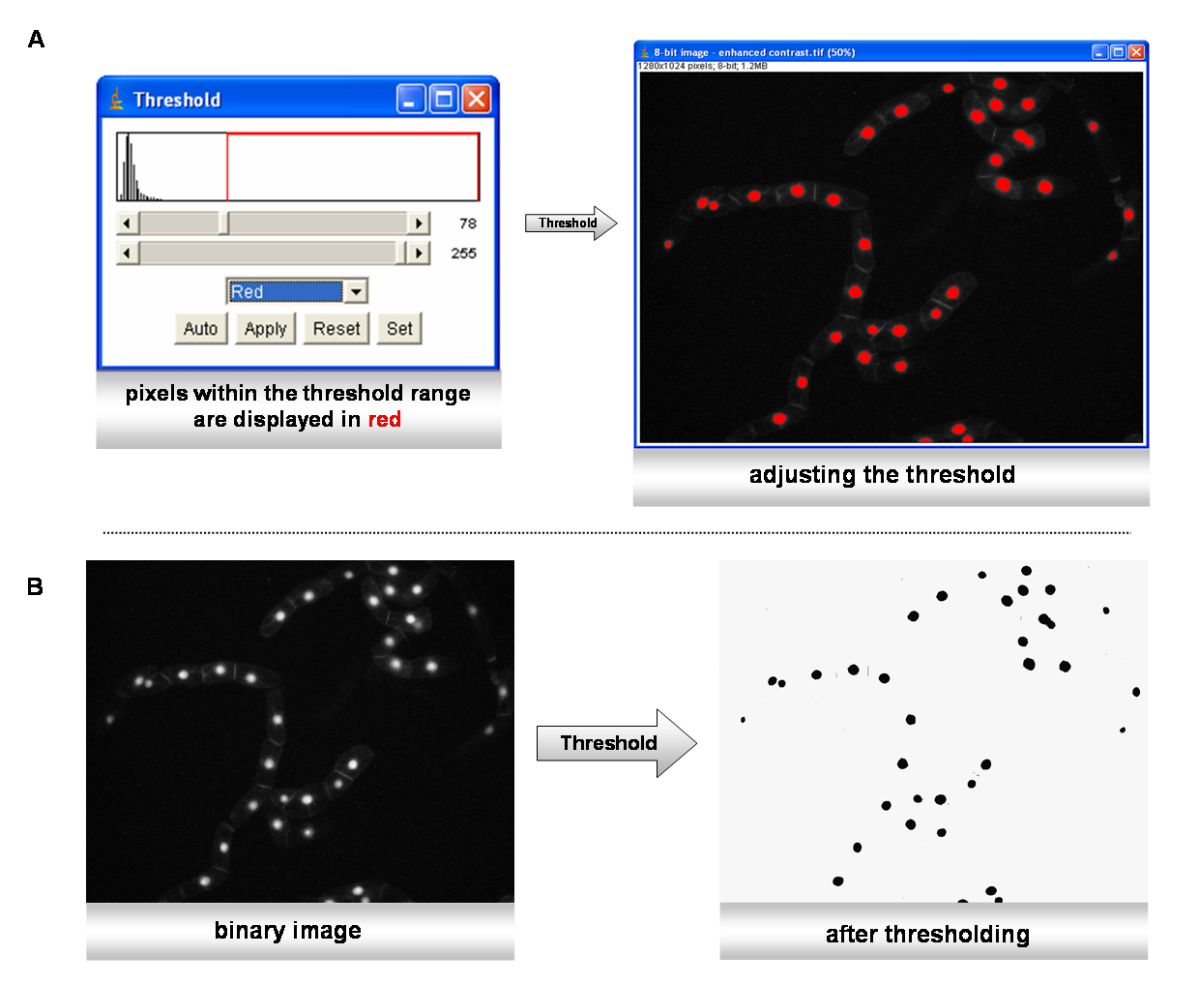
Threshold process. **A**) The threshold range can be adjusted automatically. Red pixels show objects that are within the threshold range. **B**) After pushing the “apply” button, the image is converted to black and white.

***Thresholding***. The image requires conversion into a “binary” image, for instance into black and white ( Figure 2). This can be achieved by using the menu command “Image/Type/8-bit”. Depending on the image parameters, such as luminosity and brightness, the contrast may be enhanced, with “Process>Enhance Contrast” ( Figure 2). The thresholding is essential, as the software has to discriminate between the background and the borders of the object to be analyzed. The principle is quite simple: a threshold range is set, either manually or automatically, and all the pixels within this range are converted to black and those with values outside this range, converted to white (colors can be inverted). During this process, the pixels within the range are displayed in red, whereas the background remains black. By moving the scroll bars in either direction, pixels at the periphery of objects are added or deleted from the image, as demonstrated in Figure 3.

Automatic thresholding (GFP-BD-CVIL BY-2 cell line treated with 30 µM OC) resulted in removing most of the pixels related to membranes. Only the strongest signals coming from the fluorescent nuclei remained visible. This step was particularly important as it removes the problems of user-generated bias. To guarantee maximum reproducibility, the MBF ImageJ bundle comes with a whole collection of plug-ins, using different algorithms for image thresholding ( Figure 3).

***Segmentation process - watershed separation***. When using cell suspensions in microscopy, it is almost impossible to obtain images without overlapping cells or cells clumped and clustered into small groups, even if the dilution of the cells has been optimized beforehand. To minimize counting errors due to these problems, such as (in our case) nuclei that are slightly in contact with each other, watershed separation was used (“Process>Binary>Watershed”). The principle of watershed separation is quite simple ^85^. It is based on the so-called “Euclidian Distance Map” (EDM). Any black pixel in the image is replaced with a grey pixel, whose intensity is proportional to its distance from the next white pixel. The intensity increases the closer it gets to the center of the object. In summary, this algorithm erodes objects from a binary image until they disappear. Then, it dilates them back, until they touch another black pixel. At the point of contact, a watershed line is drawn ( Figure 4a).

**Figure 4a.**
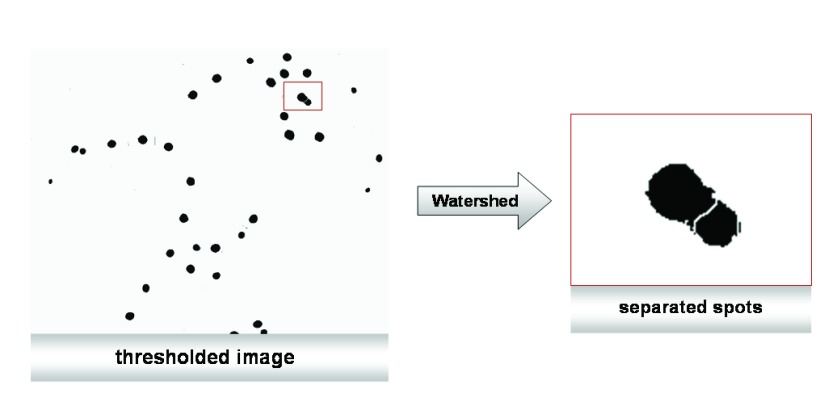
Overlapping items (fluorescence-labeled nuclei) can be separated using the watershed method. The image from Figure 3 contains nuclei that are localized so closely together that they need to be separated by the watershed method describe in the main text.

***Particle analysis***. There are several different options for counting particles with ImageJ. The menu command “Plug-ins>Particle analysis” shows a variety of plug-ins coming with the MBF ImageJ bundle. Among those are the “cell counter” and the “nucleus counter” plug-ins. In the context of our screening system, the default particle-counting menu “Analyze/Analyze particles” proved to be sufficient.

The desired parameters for particle-counting could easily be adapted for this purpose. By setting the minimum size of the object and the degree of circularity to a certain level, all objects not corresponding to the selected requirements would be excluded. Other menu points concern the visual output of the results in graphical or tabular form. The submenu routine “Show: Outlines” for example allowed to display the outlines of the detected objects with a reference number ( Figure 4b).

**Figure 4b.**
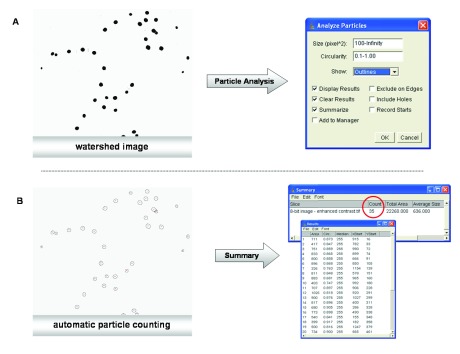
Automatic nucleus-counting with ImageJ. **A**) Particle analysis; **B**) Automatic particle-counting based on the data generated by the watershed method.

The “nucleus counter” plug-in mentioned before summarizes and automates many of the steps discussed before. Optionally, a summary of the results can be displayed and exported to other applications such as Microsoft Excel. In addition, ImageJ offers the possibility to create macros with the integrated macro editor (“Plugins>Macros>Record or Edit”). This feature allowed organization of the entire segmentation and particle analysis process by creating a customized step-by-step analysis pipeline.

***Limitations of the test system***. As outlined above, detecting and counting cells that display a shift of GFP localization to the nucleus, for instance after a specific inhibitor treatment, is achievable with modern image analysis software like ImageJ. Nevertheless, these results also suggested that in order to get a statistically reliable picture of inhibitor treatment effects, it was necessary to develop a quick and inexpensive method to also detect those cells being unaffected by treatments. But, in contrast to other model systems like yeast or bacterial cells, tobacco BY-2 cells display a great variety of phenotypes in a given population of cells, such as significantly different sizes and shapes. In addition, they grow in files (optimally in tetrades, four cells in a row) or clusters. On a regular microscope slide these tend to overlap depending on the dilution of the cell suspension, which makes automatic detection/recognition of individual cells and features within them and their correct attribution extremely challenging with the software solutions available to us. Of course, there are several possible solutions, including the development of a custom-made algorithm, but this is usually very time-consuming and involves the work of software experts, as well as a whole cascade of follow-up validation experiments.

At this point we also thought about fixing the cells before examination by standard protocols, though this is inconvenient for monitoring a treatment over a certain period of time. A direction that has not been explored for eliminating at least the problem of heterogeneous cell shapes, is the generation of protoplasts and their analysis by flux cytometry. However, this approach was not considered here because the cell wall digestion might trigger a massive stress response in cells, which could have a direct effect on the localization of the fluorescent reporter protein H _6_-GFP-BD-CVIL. For instance, we observed that cells that were exposed to different stress factors (high temperatures, no agitation over-night) were less sensitive to inhibitor treatments. Preliminary tests showed that addition of methyl jasmonate (MeJA) to cells treated with 40 µM OC could partially complement the inhibitor effects, suggesting that different hormones might influence the pool of prenyl diphosphates in response to biotic or abiotic stress.

Therefore, we decided to choose a less crude approach for counting untreated cells and to stain the nucleus with another fluorophore, as this technique had already proved valuable for the detection of inhibitor-treated cells. This approach should theoretically allow easy determination of the ratio of affected (GFP fluorescence in the nucleus) to non-affected cells (GFP fluorescence mainly localized at the PM). However, none of the commercially available and well-described nuclear stains (DAPI (4'-6-diamidino-2-phenylindole), Hoechst 33342 and 33268, etc.) worked satisfactorily with our model system.

### Optimization of the assay - inducible nuclear staining of the cell line (NLS-mRFP)

Due to the difficulties in efficiently staining nuclei in living tobacco BY-2 cells, a new strategy was chosen, consisting of transforming the existing GFP-BD-CVIL BY-2 cell line with an estradiol-inducible vector ^86^, thereby driving the expression of a red fluorescent protein (RFP), C-terminally fused to a nuclear localization signal (NLS). The goal was then to stably transform the cell line (H _6_-GFP-BD-CVIL) with a second, inducible gene construct, which specifically stains the nuclear region of BY-2 cells. Therefore, the NLS sequence of the large simian virus (SV) 40 T-antigen (PPKKKRKV) known to be sufficient for targeting several proteins to the nucleus of mammalian and plant cells ^87– 92^ was fused N-terminally to the coding sequence of mRFP (monomeric RFP) and placed under the control of an estradiol-inducible promoter in the pER10 vector system ^86^. The resulting vector was then used to stably transform the H _6_-GFP-BD-CVIL cell line via Agrobacterium-mediated transformation as described before ^30^. Lee *et al.* ^93^ showed that transformation of tobacco protoplasts with a *SV40::RFP* construct resulted in an efficient targeting of the fusion protein to the nucleus, making this protein a promising candidate. For the co-localization studies, we had chosen an enhanced RFP described by Campbell *et al.* ^94^. The mRFP is a substantially modified version of the RFP from *Discosoma* coral (DsRed or drFP583 ^95^), which was improved in regard to many major required characteristics, especially in the context of dual color imaging with GFP, one of the most important aspects relevant to a visual test system. For instance, the enhanced fluorophore mRFP is stable and bright, has a significantly shorter maturation time and its emission peak occurs at approximately 607 nm, thus facilitating the optical separation from the emission of the sGFP (synthetic GFP ^96^, which is a codon-optimized version of the green fluorescent protein (GFP) from *Aequorea victoria* ^97– 100^, with an emission peak at 511 nm ^101^.

***The N-terminal fusion of the SV40-NLS peptide efficiently targets mRFP to the nucleus of the double transformed tobacco BY-2 cell line***. To investigate if the NLS fusion protein localizes to the nucleus and can be properly co-expressed with the GFP fusion protein of the pTA7001-H _6_-GFP-BD-CVIL line, 3-day-old BY-2 cells were transformed with the pER10-NLS-mRFP vector by agroinfection. (The original pER10-NLS-mRFP vector was kindly provided by Prof. Nam-Hai Chua, Rockefeller University, New York.) Calli were selected on BY-2 solid medium supplemented with 30 µg/ml hygromycin and 50 µg/ml kanamycin (Sigma). First calli appeared after 3 to 4 weeks of growth in the dark at 26°C and were subcultured twice on solid medium until liquid pre-cultures (10 ml with the same two antibiotics) were started. These pre-cultures were grown for 7 to 10 days under permanent shaking (154 rpm, routinely at 26°C, in constant darkness), until they reached a suitable optimal density. After parallel induction with estradiol and dexamethasone for 15 h, the pre-cultures were screened visually by fluorescence microscopy. Images were acquired using the microscope settings described in Table 1 (properties of the fluorochromes and fluorescent proteins used in this work). Twelve cultures out of 36 (33.3%) showed cells expressing both fluorescent proteins.

**Table 1.** Properties of the fluorochromes and fluorescent proteins used in this work.

Fluorochrome	Purpose	Excitation peak (nm)	Emission peak (nm)	Laser (excitation)	Filter settings
**DAPI (4',6-diamidino-** **2-phenylindole,** **dihydrochloride)**	DNA staining (visualisation of nuclei)	358 (bound to DNA)	461 nm (bound to DNA)	Diode laser (405 nm)	“long pass” (475 nm)
**Hoechst 33342**	DNA staining (visualisation of nuclei)	350 (bound to DNA)	461 nm (bound to DNA)	Diode laser (405 nm)	“long pass” (475 nm)
**Propidium Iodide**	DNA staining (visualisation of dead cells)	535 nm (bound to DNA)	617 nm (bound to DNA)	Helium-Neon laser (543/633 nm)	“band pass” (575–615 nm)
Fluorescent Protein	Purpose	Excitation peak (nm)	Emission peak (nm)	Laser (excitation)	Filter settings
**sGFP (synthetic Green** **Fluorescent Protein)**	intracellular localisation of fusion proteins	488 nm	511 nm	Argon laser (488 nm)	“band pass” (505–550 nm)
**mRFP ( monomeric Red** **Fluorescent Protein)**	intracellular localisation of fusion proteins	584 nm	605 nm	Helium-Neon laser (543/633 nm)	“band pass” (575–615 nm)

**Figure 5.**
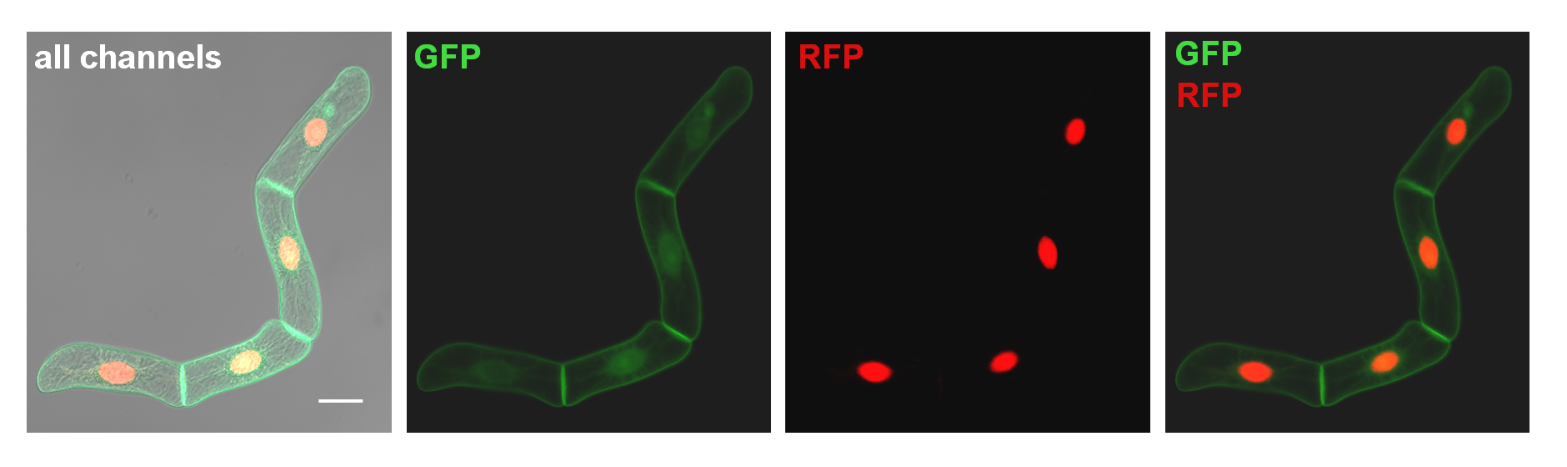
Double-transformed BY-2 cell line (N-20) showing different intracellular localization of fluorescent fusion proteins (after induction). **All channels:** tetrade with red and green fluorescence induced at the same time (+ differential interference contrast, DIC). **GFP:** the GFP-BD-CVIL fusion protein is visualized after induction with 10 µM Dex and is mainly localized to the PM and cytosol. **RFP:** The monomeric RFP fused to the C-terminus of the SV40 NLS is visualized after induction with 5 µM estradiol and is predominantly found in the nuclear compartment. **GFP/RFP:** Overlay of the GFP and RFP channels. Induction time with both elicitors was 15 h. Images were acquired using a LSM510 confocal laser scanning microscope equipped with an inverted Carl Zeiss axiovert 100 M microscope. Dual color imaging was performed using dual excitation/emission scanning in the multitracking mode (Carl Zeiss Laser Scanning Microscope software). White bar represents 20 µm.

The most promising cell line (N-20) showed bright fluorescence in both channels set for the visualization of GFP and RFP, respectively, and had a significant ratio of fluorescent positive cells (>50% of total cells), making it a good candidate for first tests and further re-selection efforts to obtain a performing cell line suitable for statistical approaches. In Figure 5 a typical cell tetrade is displayed, after induction by both dexamethasone (Dex, green fluorescence) and estradiol (Est, red fluorescence).

**Figure 6.**
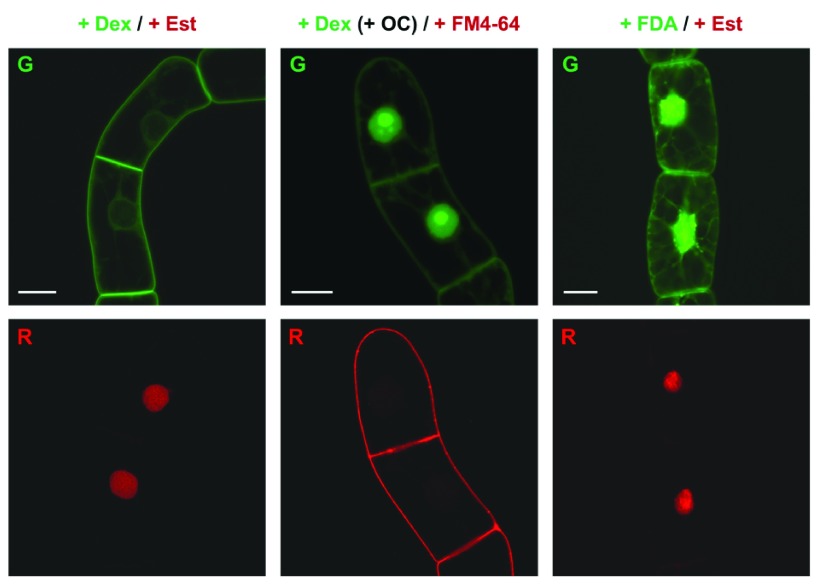
Expression of fusion proteins is tightly regulated by their specific inducers. No cross-induction of fusion gene expression was visible. Induction time for dexamethasone (Dex, 10 µM final) and estradiol (Est, 6 µM final) was 15 h in all experiments. **+ Dex / + Est:** control experiments with both inducers with the GFP fusion protein targeted to the PM (G) and the mFRP fusion protein located in the nucleus (R). **+Dex (+OC) / + FM4-64:** Dex alone is only inducing the expression of the GFP fusion protein (G). The negative control with FM4–64 (5 µg/ml; membrane stain) shows no signals from the nucleus (R). For a better understanding, cells were treated with 50 µM oxoclomazone (OC) 3 h before induction, to indicate the position of the nucleus. Fluorescein diacetate ( **FDA**, 7.5 µM final) is used as negative control, also indicating that the cells were alive during the imaging process. **G** and **R** indicate the green and the red channel, respectively. Bars = 20 µm.

***The newly created double fluorescent cell line NLS-mRFP/H _6_-GFP-BD-CVIL shows no visible cross-induction of fluorescence after treatment with estradiol or dexamethasone***. In order to establish a reliable visual test system, it was necessary to verify that the two co-existing induction systems in the NLS-mRFP (Est) and H _6_-GFP-BD-CVIL (Dex) lines only respond to their specific inducers. As both chemical-inducible systems are based on a similar principle of induction - the action of chimeric *trans-activators* whose transcriptional activities are regulated by specific hormones and/or structurally related compounds ^86^) - the newly generated cell line was separately treated with both inducers under standard conditions. In addition, fluorescent cell dyes were used in parallel as negative controls ( Figure 6). Simultaneous treatment of the cell line with both inducers (24 h induction, 10 µM Dex, 6 µM Est) resulted in a phenotype with green fluorescence predominantly located at the peripheral membrane region and red fluorescence mainly located in the nuclear compartment.

After treatment with Dex only (24 h, 10 µM), the observed cells did not show any detectable signal in the red channel (RFP). As a negative control, these cells were treated with the plasma membrane stain FM4-64 (5 µg/ml, 5 min treatment). FM4-64 fluorescence was clearly visible in the red channel ( Figure 6), whereas no detectable signals appeared in the nuclear region (the cells had been treated with 50 µM OC to indicate the position of the nucleus).

Cells treated with Est only (24 h, 6 µM) showed an accumulation of red fluorescence in the nuclear region and no detectable green fluorescence. As a negative control, the viability cell stain fluorescein diacetate (FDA ^102^) was used (7.5 µM final, 2 min). Green fluorescence could be observed in the whole cell, which also indicated that the cells were living when the image was taken ( Figure 6).

***24 h are sufficient to obtain homogenous red fluorescence***. Another important aspect was the optimal duration of the induction times for both fusion proteins, in order to determine at which time point the intensities of the emitted signals were sufficiently strong and homogenous for detection and image acquisition. To address this issue, the cellular localization of the NLS:mRFP reporter protein was examined at various times after induction with Est by fluorescence microscopy. 7-day-old cells were diluted 6-fold in MS-BY-2 medium and 3 ml were dispatched into the wells of a 6-well plate. These cells were then cultured at 26°C in obscurity and under permanent shaking. Induction with 6 µM Est (solubilized in benzene/ethanol, 1:1) took place at 48, 36, 24, 18, 15, 12, 6 and 3 hours before observation. The settings for image acquisition in the red channel did not change during the whole experiment.

**Figure 7.**
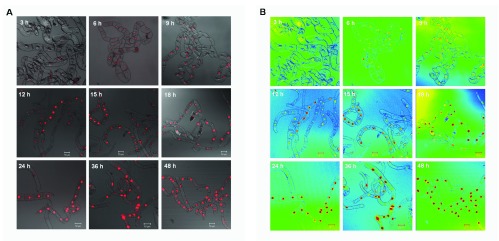
Time course of induction for the NLS-mRFP. *In vivo* targeting of the NLS-mRFP fusion protein after induction with 6 µM Est (and in the presence of 10 µM Dex – not shown). The red fluorescent signals were examined at different time points after induction. **A**) Overlay of transmission microscopy images with the RFP channel. **B**) Same images as in **A** shown in LSM image browser rainbow mode (red indicates saturated areas). Saturation is already reached after 24 h of induction with both elicitors. All images were acquired with the same microscope settings (red channel, Carl Zeiss LSM510 microscope; EC-Plan-Neofluar, 10x/0,3 M27)

To determine the level of saturation, images were converted to a rainbow scale with the Zeiss LSM image browser. Red signals indicate saturation. As seen in Figure 7[A], nuclear localization of the NLS:mRFP protein could already be observed 3 h after induction. Nevertheless, it took at least 24 h until most of the signal arising from the nuclei was saturated (red dots, Figure 7[B]).

**Figure 8.**
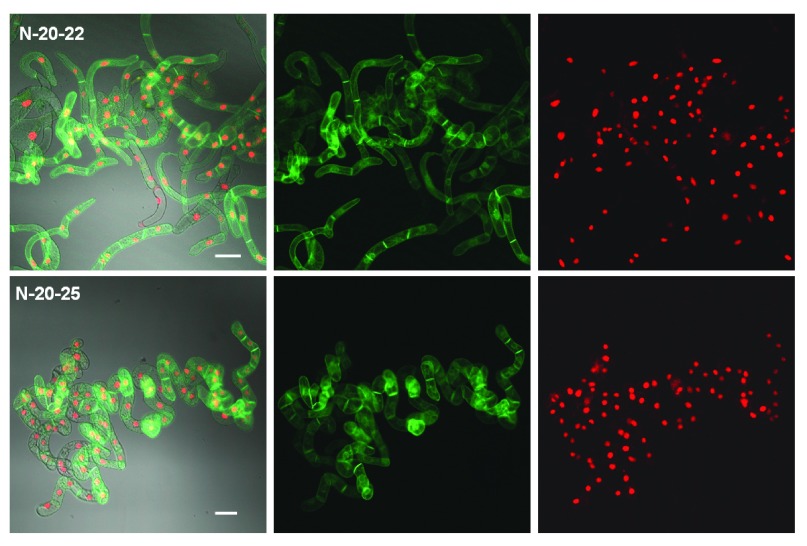
Two different clonal selections of the double-transformed cell line N-20. The most promising cell line N-20 was re-selected in order to obtain a performing cell line. The resulting cell lines (N20–22 and N20-25) are characterized by a high ratio of bright fluorescent cells (>95%). Images were taken as described in the legend to Figure 5. Cells were induced for 24 h with 10 µM Dex and 6 µM Est (final concentrations). White bars represent 50 µm.

***Cloning of transgenic BY-2 cells: generation of a cell line with strongly reduced heterogeneity***. Generation of a performing double-transformed cell line was a process of constant engineering of the initial GFP-BD-CVIL BY-2 cell line in order to obtain a clonal selection of cells responding appropriately to different stimuli. In the course of this procedure, cloning of primary heterogeneous suspensions generated secondary homogenous lines. The resulting calli and suspensions derived thereof were both screened and evaluated by fluorescence. In this context it is important to consider that the terms “homogenous” and “heterogeneous” do not describe the intensity of the fluorescence, but rather if a given culture showed well-balanced and stable fluorescence within the cell population. To achieve this goal, the most promising double-fluorescent cell line (N-20) was chosen for a rigorous re-selection process. Two liquid cultures started from these secondary calli showed bright fluorescence in both channels as well as a high ratio of fluorescent cells (>95%) and were maintained for further experiments ( Figure 8). Nevertheless, it proved to be a time-consuming challenge, as within cell suspensions of supposedly clonal origin (primary suspensions derived from primary calli), important variations were regularly observed. These variations not only concerned the morphology of the cells, but also more importantly the homogeneity and intensity levels of the fluorescence. Therefore, a major focus was to re-select homogenous transgenic cell lines with high intensity levels of fluorescence. A relatively simple method to generate more homogenous cell lines, derived from secondary calli, was established and constantly improved over time, leading to the final protocol summarized in Table 2 together with a comparison of a procedure recently published by Nocarova and Fischer ^103^.

**Table 2.** Comparison of the cloning procedures of transgenic tobacco BY-2 cells.

Cloning procedure	Nocarova and Fischer, 2009	This paper
**Transformation method**	*Agrobacterium tumefaciens*	*Agrobacterium tumefaciens* LB4404
**culture preparation from** **primary calli**	1 ml of fresh calli in 30 ml medium (+AB) by pipeting	small calli (diameter ~5 mm) in 10 ml medium (+AB) with a spatula
**subculturing of primary** **suspensions**	after 7 days: 1,5 ml in 30 ml medium	after 7 days: 1–2 ml in 40 ml medium
**cloning of secondary calli**	7-day-old transgenic cells are diluted 1:3 and mixed with 4ml of similarly prepared WT cells in a ratio 1:1000	7-day-old WT cells are diluted 1:10 (3 ml + 27 ml of medium) and mixed with 7 day old transgenic cells (60 µl) in a ratio 1:500
**dilution factors and ratios**	7-day-old wild-type: 1:3 7-day-old transgenic cells: 1:3 transgenic:wild-type: 1:1000	7-day-old wild-type: 1:10 7-day-old transgenic cells: - transgenic:wild-type: 1:500
**Spread on solid medium** **(+AB)**	500 µl on Ø 6 cm Petri dish	7 ml on Ø 12 cm Petri dish (sufficient to cover the surface)
**calli appearing (after 3–6** **weeks)**	~25	~25 – 100

This method proved to be quite efficient, as we succeeded in obtaining several secondary suspension cultures of the double-transformed mRFP-cell line that exhibited a high percentage of bright fluorescent cells (> 95%) under our experimental conditions.

### The problem of putative DNA methylation events explaining some heterogeneity of transformed BY-2 cells

**Figure 9.**
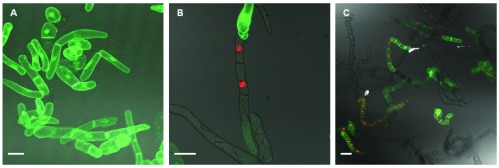
Restoration of RFP fluorescence after treatment with 5-azacytidine. **A**: Double fluorescent cell line that suddenly lost mRFP expression. **B** and **C**: Cells were treated with 10 µM azacytidine for one week. RFP fluorescence could be recovered, but many dead cells were due to overall toxic effects. Even though the concentration was scaled down 10–20x compared to values that are indicated in the literature for treatments, the concentration still seemed to be too high for the use in tobacco BY-2 cells (which have a good uptake rate and fast metabolism). All images are shown as merged images taken in green and red fluorescence, as well as white light mode. White bars = 50 µm.

After some problems with the maintenance of culture conditions due to a failure of the temperature control system of the growth chamber, we observed a sudden loss of all the mRFP fluorescence in one of the double-transformed cell lines, whereas the level of GFP fluorescence remained completely untouched ( Figure 9). After elimination of all evident sources of error (replacing the inducer, subculturing the two-week old line, replacing the medium), the culture was incubated over a whole 7-day-growth cycle in presence of 10 µM 5-azacytidine, a nucleotide analog that cannot be methylated and, remarkably, the mRFP fluorescence could be partially restored ( Figure 9). As this result clearly suggested a DNA methylation event, we screened the literature for common sources of such sudden drops in gene expression levels of transgenes in plant cultures. As we had already observed the same phenomenon in the original H _6_-GFP-BD-CVIL cell lines during the inhibitor tests with fosmidomycin-derived prodrugs, this point was quite important, given all the effort put into the generation of the cell lines. Schmitt *et al.* ^104^ reported that the antibiotics kanamycin, hygromycin and cefotaxim caused a DNA hypermethylation at CpG sites in the genome of tobacco plants grown *in vitro,* as shown by the SssI methylase accepting assays and genomic sequencing with sodium bisulfite. Interestingly, these methylations occurred in a time and dose-dependent manner and were not reversed when the progeny was not grown anymore in the presence of the antibiotics ^105^.

The methylation of plant genomes is a common process, which can affect up to 30% of the cytosine residues ^106^. It also occurs as part of “natural” gene regulation in plants ^107, 108^. However, increased DNA methylation was observed in several cases associated with PTGS (post-transcriptional gene silencing) and TGS (transcriptional gene silencing) or different forms of stress ^109– 112^. For instance, transgene silencing was induced in *Petunia*, after a period of high light intensity and temperature ^113^, whereas high temperatures alone were shown sufficient to silence different transgenes in tobacco ^114– 116^. Thus, major breakdowns of the air-conditioning system of our growth chambers (several weeks during summer, at > 27°C), might well have contributed to the observed silencing. However, although recovery of the mRFP expression by azacytidine treatment had confirmed our assumption that the sudden loss of fluorescence, due to a DNA methylation event, could explain the heterogeneity of the cells, we did not maintain and subculture the recovered lines, as azacytidine is a powerful mutagenic agent ^117^, which might exert pleiotropic effects on the cells.

***Response of the doubly transformed cell line to various treatments***. As the major emphasis of this project was the development of a tool to screen for inhibitors of the MEP pathway and the prenylation reaction itself, it was necessary to verify that the newly generated cell line showed the same phenotype in response to various treatments as the initial cell line H _6_-GFP-BD-CVIL despite the presence of a second, inducible fusion protein.

**Figure 10.**
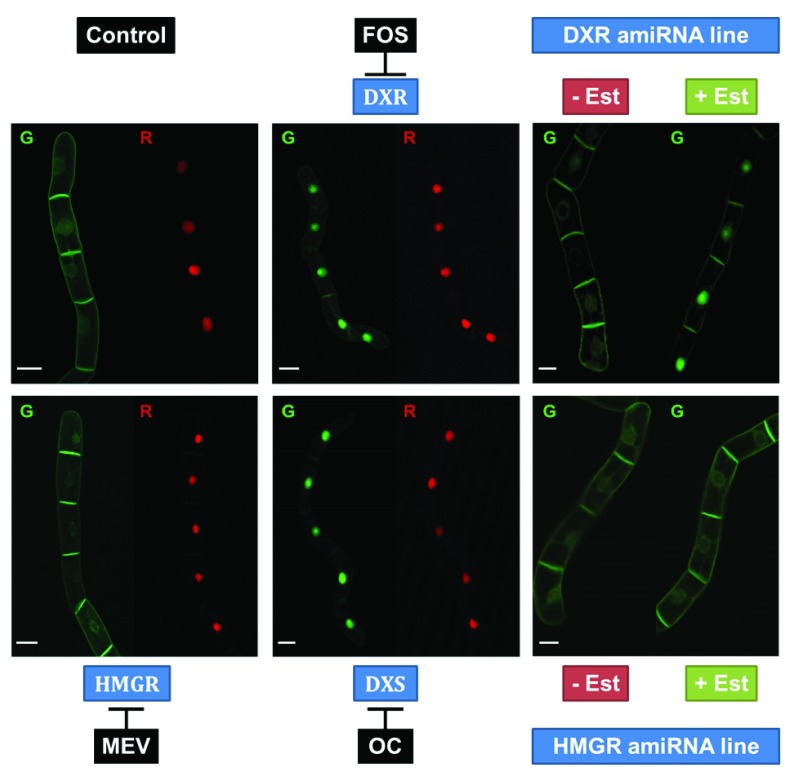
Two-channel imaging of the N-20 cell line after various treatments. The cell line was treated with different inhibitors affecting the **MVA pathway** (mevinolin) and the **MEP pathway** (fosmidomycin, oxoclomazone). Induction of the cell line with Est and Dex was carried out 24 h before observation. The cell line was treated with specific inhibitors 18 h before observation. Fosmidomycin ( **FOS**) and oxoclomazone ( **OC**) clearly shifted the localization of the GFP fusion protein to the nucleus, whereas mevinolin ( **MEV**) treatment had no visible effect on the cells. In addition, we used two new cell lines with artificial micro interfering ( **amiRNA**) silencing constructs as “biological controls”. (These cells expressed only the GFP reporter protein under the control of the dexamethasone-inducible promoter besides the amiRNA constructs.) For instance, **DXR**-silenced cells show the same phenotype as those inhibited *in vivo* by Fos. **HMGR** silencing however does not exert any visible effect on the localization of the GFP fusion protein. White bars represent 20 µm.

The doubly transformed BY-2 cell line was treated for 18 h with inhibitors like oxoclomazone (OC), fosmidomycin (FOS) and mevinolin (MEV) affecting key enzymes of the MEP and MVA pathways, respectively, as indicated in the legend to Figure 10. Induction with 10 µM Dex and 6 µM Est took place 24 h before observation ( Figure 10).

As expected, inhibition of the cytosolic MVA pathway by mevinolin did not show any effect on the predominant localization of the green fluorescent fusion protein at the periphery of the treated cells, which is very similar to the fluorescence pattern of untreated cells. In contrast, inhibition of the first two enzymes in the MEP pathway (DXS and DXR) by OC and FOS at 40 µM final concentration each resulted in the previously described, nearly complete translocation of GFP-BD-CVIL to the nucleus, which is consistent with the results obtained in our previous study ^30^. In all cases, the NLS-mRFP protein could be induced and co-expressed without affecting the expression or localization of the GFP-BD-CVIL protein.

**Figure 11.**
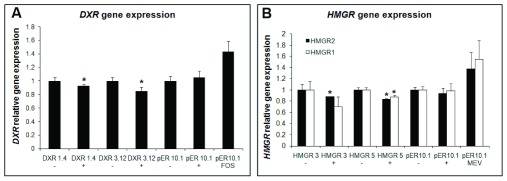
DXR and HMGR gene silencing via inducible amiRNA in BY-2 cells. Two Nicotiana tabacum BY-2 lines, independently transformed with inducible amiRNA constructs against DXR (DXR 1.4 and DXR 3.12) or HMGR (HMGR 3 and HMGR 5) were treated 1 day after sub-culture with (+) or without (-) 5 μM estradiol. Control lines were also treated with FOS or MEV. Gene expression levels for DXR ( **A**), HMGR1 and HMGR2 ( **B**) were measured by qPCR after 24 h of treatment. Experiments and measurements were repeated three times. Statistically significant changes are indicated (asterisk, p-values<0.05). Changes in expression in a BY-2 control line (transformed with the empty vector, pER10.1) are also shown.

As a further proof of concept, different cell lines were generated, targeting *HMGR* (MVA pathway) and *DXR* genes (MEP pathway) with an artificial micro-RNA (amiRNA) strategy ^118^ in order to confirm the effects of the inhibitors on their target proteins on a biological level ( Figure 10). DXR-silenced cells exhibited a phenotype similar to FOS and OC treatment, whereas the silencing of HMGR did not have any effect on the localization of GFP-BD-CVIL, similarly to what happens with MEV addition. Expression levels for *DXR* and *HMGR* were tested and, as expected, lines treated with FOS showed increased expression of *DXR* whereas lines treated with MEV showed *HMGR* overexpression. When gene expression was assayed in amiRNA lines, in both cases, the levels of silencing reached were not very high (10–15% for DXR and 10–30% for HMGR, Figure 11) and were in the same line as levels achieved when silencing cycloartenol synthase, an enzyme involved in sterol biosynthesis ^119^. These results could be explained by recent observations on the dependency of miRNA formation on the biosynthesis of sterols: Apparently the formation of repressive complexes with ARGONAUTE (AGO) proteins needs a membrane association in which sterols are functional ^120^, as was shown with miRNA action-deficient ( *mad*) *Arabidopsis* mutants 3 and 4. *MAD3* encodes HMGR1, and *MAD4* encodes sterol C-8 isomerase, catalyzing an important step in cytoplasmic sterol biosynthesis ^120^. Thus, silencing of a gene coding for a key enzyme like HMGR or downstream in the pathway might be difficult to achieve beyond a rather low degree ^120^.

**Figure 12.**
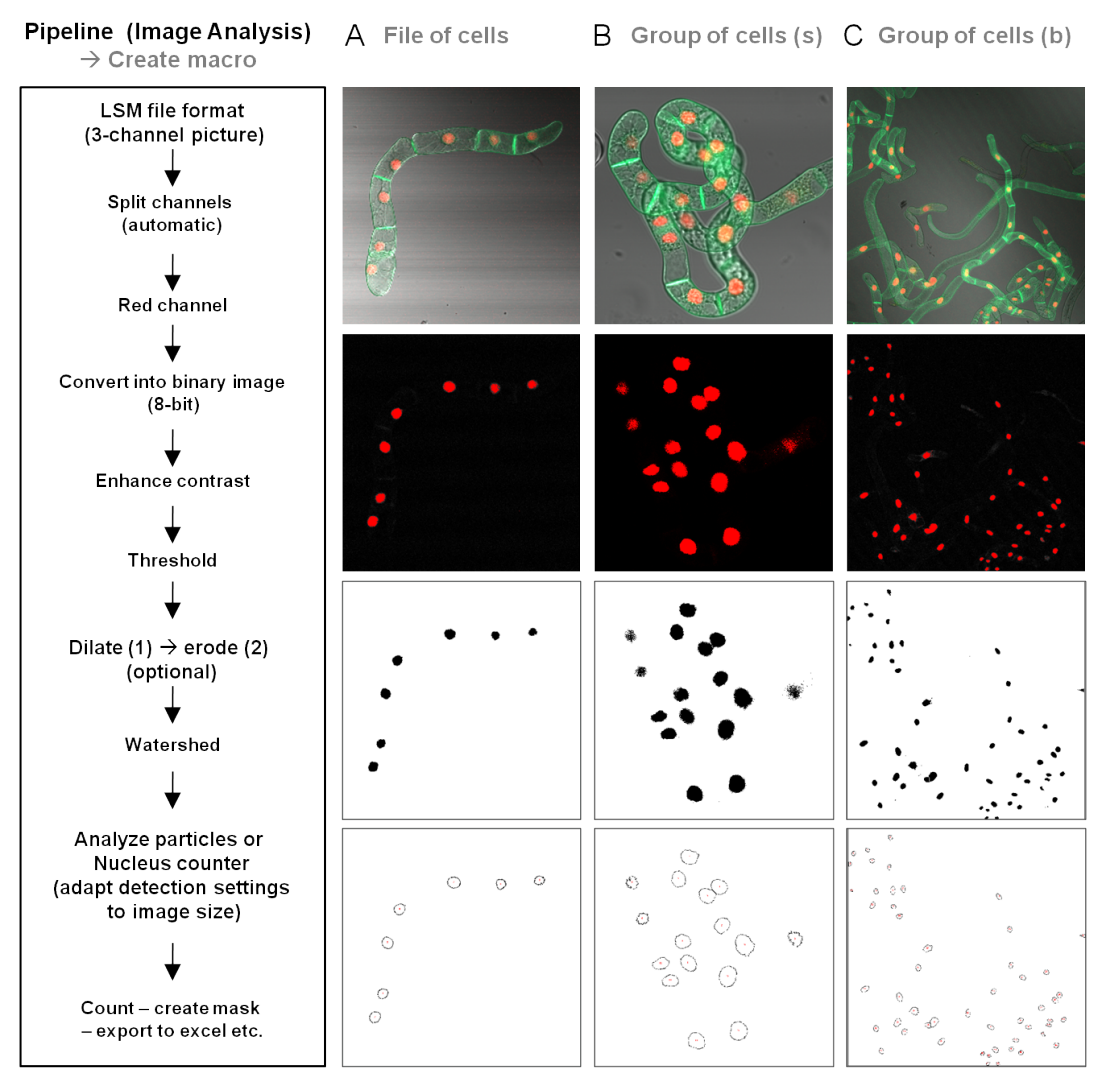
Use of the mRFP fluorescence (red channel) to detect and count BY-2 cells. The flow of actions to be taken is shown left, and corresponding images and their conversion for particle counting is shown for **A**) a file of cells, **B**) a group of cells at higher magnification, and **C**) at lower magnification.

***The mRFP fluorescence can be used for the identification/quantification of cells***. The proposed approach to determine the total number of fluorescent tobacco BY-2 cells in an image was to stain the nucleus, as this technique was successfully applied to identifying cells that showed a mislocalization of the GFP fluorescence to the nucleus in response to the treatment with the MEP pathway inhibitor oxoclomazone. Figure 12 shows and explains three different scenarios for the detection of cells, ranging from selected cells to whole-population images.

To obtain statistical data, we combined the analyzed images of the red and green channels. The detected nuclei in the red channel are used as a mask that serves as a landmark for the zone we intend to investigate in the green channel, the nucleus ( Figure 12). This proved to be a promising approach for the analysis of rather simple scenarios and its applicability for more complicated scenarios has to be further investigated. Critical points are the homogeneity and intensity of the fluorescence. This protocol can however be saved and re-used for the quick analysis of multiple images or sets of images, given that the image acquisition settings are identical. Therefore, the more constant the treatment conditions, the fewer follow-up adjustments for the image analysis that are required.

***The mRFP fluorescence emitted at the nucleus can be used to find the optimal focal plane for the acquisition of images in double fluorescence mode***. Modern image-based screening approaches typically use multi-well plates for efficiently screening chemical libraries, at a medium to high throughput level ^38, 56, 64^. This requires the acquisition of several images from every well, a task that is commonly accomplished by automated microscope platforms. One of the major challenges of these systems remains the focusing technology ^121^. Depending on the application, individual routines often have to be developed in order to acquire images of adequate quality for later image analysis. Fluorescence-based focusing has several disadvantages, including photobleaching and possible phototoxicity ^122, 123^. However, observations made during manual focusing with the double-fluorescent cell lines indicated that the maximal intensity in the red channel (nuclear-localized mRFP) correlated with the focal plane, found by a human observer. These early results suggested that the fluorescence emitted by the mRFP could be used for later autofocusing purposes, keeping in mind that one of the main features we are interested in is the change of subcellular localization of the prenylatable GFP fusion protein from the periphery of the cell to the nucleus after inhibition.

**Figure 13.**
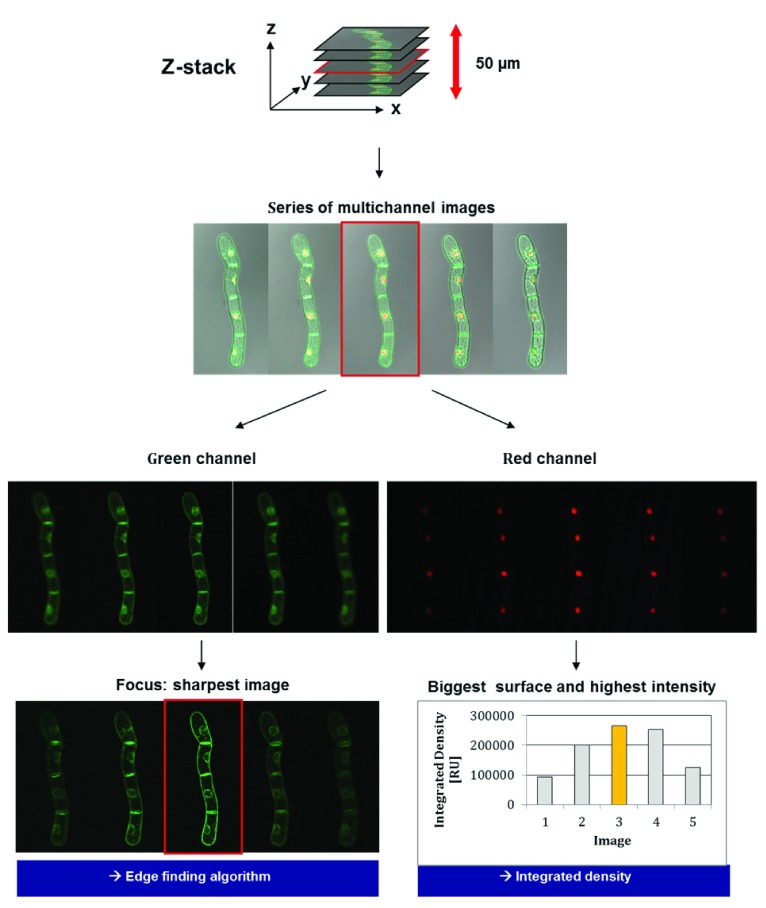
Correlation between the focal plane and the strongest signals in the red channel. Images are subsequently taken at different levels of the sample (Z-stack). The optimum sharpness of signals for RFP indicates the focus being set on nuclei. The determination of the sharpest image for the GFP signal provides an integrated optimum for the analysis of doubly transformed BY-2 cells.

To confirm these early observations, a series of multichannel images of BY-2 cells (expressing both fluorescent proteins) spanning a total distance of 50 µm in the Z-plane was acquired at different focal planes. Afterwards, each optical slice of this Z-stack was analyzed using ImageJ software ( Figure 13). The images of the green channel were analyzed by the edge-finding algorithm of ImageJ, whereas the integrated density was calculated for the red channel. The results clearly show that, for the green channel, the sharpest image (as perceived by a human observer) of the Z-stack (identified by the edge-finding algorithm of ImageJ) is also the image with the highest integrated density in the red channel, which is defined as “the sum of the values of the pixels in an image or selection” ( ImageJ online manual). This correlates very well with general observations about fluorescence images that indicate a maximum image contrast at the Z-stage height corresponding to the focal position ^122, 123^. Therefore, a fluorescence-based autofocusing approach could use the nucleus-located maximum of red fluorescence to define the plane of focus and acquire additional pictures at an offset from this position (in both directions). This vertical series of images could then be summed up into a single projection or used to choose the best focal plane for each fluorophore. In an optimal scenario, two different images in different focal planes should be taken, when working with different fluorophores/wavelengths, due to the chromatic aberration of optical lenses (objectives), which means that different colors/wavelength of light are focused to different points ^124^.

### Towards miniaturization and the right choice of the format

Modern microscope-based screening approaches typically use multi-well plates, as these allow a significant increase in the number of tested compounds per day and save reagents and consumables at the same time due to the miniaturization of the experimental setup ^125^.

The most common format for classical medium (up to 10,000 compounds/day) to high throughput (between 10,000–100,000 compounds/day) screening assays are 96- or 384-well plates with average working volumes of 100–200 µl and 50 µl per well, respectively. Despite the possible efficiency gains connected with these high-density formats, there are severe technical hurdles for their use in routine HTS assays, most importantly the adaptation of automated liquid handling and dispensing technology, which is better established for the bigger 96- and 384-well formats ^125, 126^.

Our goal was to significantly decrease the working volume for the assay and to use a format fulfilling the requirements of modern cellular imaging platforms, capable of (automatically) acquiring images at a (reasonable) throughput rate. However, the image quality should still be sufficiently good to monitor whole cell populations, on the one hand and to measure intracellular events on the other.

Ideally, multiple images at different positions and different magnifications should be acquired from each well. This kind of read-out however would be extremely time-consuming and take several hours to process an entire multi-well plate. Therefore, it was necessary to find the right balance between high content and a reasonable throughput or, in other words, between time, cost and quality, which Mayr and Fuerst ^125^ called “the magic triangle of HTS”.

***Criteria and parameters to be considered***. In order to find the format providing us with the greatest flexibility as far as the quality of the image acquisition, the growth conditions of the BY-2 cells, and the general liquid handling were concerned, it was first necessary to take a closer look at some of the characteristics of the model system used in the bio-assay.

BY-2 cells usually grow in files or individual cells (in the exponential growth phase), easily reaching 50 to 100 µm in length and more than 30 µm in width, with the nucleus having a diameter between 10 and 20 µm (our observations, after measuring in average several hundred cells). Previous results already indicated that the images taken in a medium to low magnification- mode (10 × objective - resolution in the µm range) could be exploited by image analysis software and provide sufficient information for the analysis of the observed phenotypes. For images acquired with the 10 × objective (EC-Plan Neofluar 10x/0.30 M27), the field of view ranges from about 900 µm × 900 µm to 1272 µm × 1272 µm (stack size: x-plane × y-plane) in the greatest possible number of configurations. A field of this size (approximately 1 mm × 1 mm) allows the study of up to 100 cells on a conventional microscope cover slide, depending on the dilution factor of the culture. In order to obtain data from a statistically significant number of cells for each treatment, images from multiple fields should be collected from each well. Typically, 96-well plates (i.e., Cellstar ^®^ Cat.-No.650 180, Greiner bio-one, Les Ulis, 91941 Courtaboeuf, France) have an internal diameter at the bottom of the well of approximately 5 mm. The diameter of the next largest format, the 384-well plate, is already significantly smaller at about 3 mm ^126^. However, considering the size of the cells and the image field, as well as the need to acquire multiple images, all formats smaller than 96-wells did not make any sense for our experimental system.

**Figure 14.**
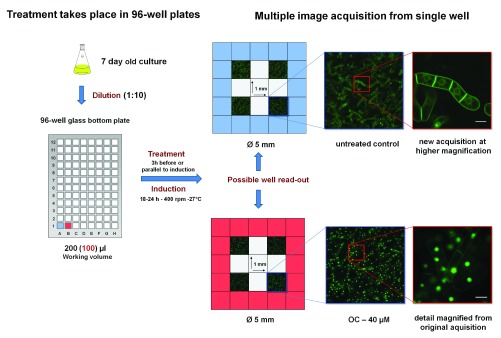
Image acquisition takes place in 96-well glass bottom plates. The scheme explains the steps of how the LSM microscope can scan wells. Images can be taken at low resolution to indicate the behavior of many cells for statistical evaluation. But at the same time high resolution is achieved by zooming into the image for detailed analysis of fluorescent protein localization, either to the PM (control) or to nuclei (after addition of an appropriate inhibitor like oxoclomazone (OC) at 40 µM).

The diameter of a conventional round-shaped well allows the acquisition of at least 9 independent fields of more than 1 mm × 1 mm, without interfering with the walls of the wells. However, one of the limitations remains the possible read-out pattern, which cannot exploit the whole surface of the well. In order to maximize the surface for the read-out, commercial square-shaped micro-array plates (with glass-bottoms) offered an interesting alternative to round 96-well plates ( Figure 14).

Figure 14 shows a field of view of 1 mm ^2^ that was acquired using an inverted fluorescence microscope (Carl Zeiss) and a 96-well plate with a glass bottom (No. 1.5, γ-irradiated, MatTek Corporation - Ashland, MA, USA) that resembles a conventional cover slide in thickness. To be able to use 96-well plates, a special stage adaptor for multi-well plates was purchased from Carl Zeiss (Invertoskop Microscope Specimen Holder). In contrast to the image acquisition for cells from a normal cover slide, the situation is a little more complex for cells in a well, as hardware and software have to cope with cells in a more extensive three-dimensional space (a suspension of cells with a height of several mms). This means that not all of the visible cells are in the right focal plane and align perfectly parallel to the z-axes. Nevertheless, this lack of “substrate flatness” ^121^ should partially be solved by the presence of a reference point for the focusing software. For that reason we generated the double-transformed cell line emitting red fluorescence through an inducible, nuclear-localization fusion protein.

Results obtained by manual focusing clearly demonstrated that it is possible to resolve subcellular details at a satisfying resolution ( Figure 14) (1024 × 1024 pixels × 8) and deliver enough information to distinguish the phenotypes of interest, here for instance the mislocalization of H _6_-GFP-BD-CVIL to the nuclei after treatment with OC ( Figure 14).

Besides the quality of the image acquisition process, another important factor for the miniaturization of the assay was the nature of the biological material, which sets distinct limits for the downscaling process and needs the adaptation of various parameters for the significantly smaller format (adjusting minimal and maximal fill volumes; agitation; minimizing the evaporation; liquid handling technology etc.)

***Problems in the optimization of culture conditions in microtiter plates***. In this context, it must be kept in mind that this bioassay, in contrast to most cell-based imaging approaches, relies on the observation of living cells. The majority of image-based screens at a high-throughput rate are usually performed with fixed cells ^64^. BY-2 cells are grown in liquid medium, which means that the treated and induced cultures require permanent shaking for more than 20 h to prevent cell sedimentation, as this may lead to sub-optimal nutrient and oxygen supply and could interfere with the expression of the fluorescent reporter proteins.

**Figure 15.**
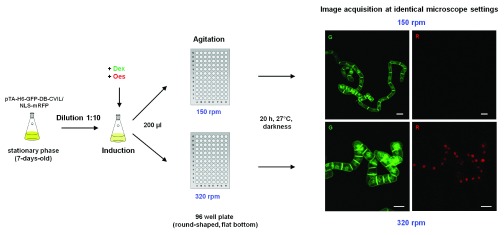
Influence of different shaking conditions. Shown is the expression of both fluorescent reporter proteins in transgenic tobacco BY-2 cells incubated over-night in the wells of a 96-well microtiter plate. A 7-day-old culture was diluted tenfold into a fresh culture medium before the inducers Dex (10 µM) and Est (5 µM) were added. Afterwards 200 µl of the cell suspension were added to the wells of a 96-well glass-bottom microtiter plate and shaken under different conditions (150 rpm and 320 rpm, respectively) before being examined by fluorescence microscopy as described in previous Figure legends. White bars = 10 µm.

We examined the influence of suboptimal agitation in this small-scale system (volume 100 to 200 µl) on the expression of the reporter proteins in several independent experiments in which different shaking conditions for the BY-2 cultures were tested. Therefore, 7-day-old cells were diluted (1:10) into fresh BY-2 medium and then induced by the addition of 10 µM Dex and 5 µM Est. Then 200 µl of this dilution was transferred into the wells of a 96-well plate (conventional round-shaped wells, with conical bottom) and incubated for 20 h in the dark under permanent shaking (160 rpm or 320 rpm). Cells that were shaken at 160 rpm (which corresponds to the shaking frequency of 6-well plates and culture flasks) showed a normal induction of the GFP fusion protein, whereas the mRFP fusion protein was barely expressed. However, in cells that were cultivated at 320 rpm, the expression of the NLS-mRFP protein could clearly be detected by fluorescence microscopy ( Figure 15). The gas-liquid mass transfer properties of shaken 96-well plates have been investigated in detail by Hermann *et al.* ^127^) and revealed that the oxygen transfer rate (OTR) measured in the wells was strongly influenced by different parameters, such as the surface tension of the medium, the material of the well, the filling volume and the shape of the well. In round-shaped wells, for example, due to the high surface tension, no liquid movement occurred until a critical shaking intensity was reached: for 200 µl of water shaken at shaking diameter of 25 mm, the rate had to exceed 300 rpm. On the other hand, frequencies above 450 rpm could not be used without the risk of the liquid spilling out of the well ^127^. As a general rule, one can say that the OTR increases proportionally with shaking amplitude and frequency due to an increase in the total surface that is available for oxygen (gas) transfer. The same effect was observed by replacing round-shape wells by rectangular or square wells, which can be explained by the increase of the turbulence of the system due to the effect of the corners. A higher fill volume on the other hand decreases the oxygen transfer rate if all other parameters are kept constant ^127– 129^. No agitation of the fluid (diluted cells in BY-2 medium) was observed for 250 µl until around 300 rpm (using a Heidolph unimax 1010 shaker, 10 mm). Therefore a frequency of 320 rpm and higher was used. However the limitation for further testing was the maximum speed of the available shaker (500 rpm). In addition, these results were obtained by using wells with a conical bottom. The use of an inverted microscope required 96-wells with a flat-bottom for the imaging process, and we found that the hydrodynamic behavior of a BY-2 culture in a flat-bottomed well differs significantly from a conventional deep-well. Preliminary results indicate that the speed has to exceed 500 rpm to assure an optimum agitation of the cells for a filling volume of 200 µl. This result prompted us to purchase 96-well glass-bottom plates with a square-shaped cross-section area/ground profile. Besides increasing the OTR at lower shaking frequencies compared to round wells, it should also confer an additional advantage to the read-out process by significantly increasing the total surface area of the well.

### Towards the automatization of the assay

**Figure 16.**
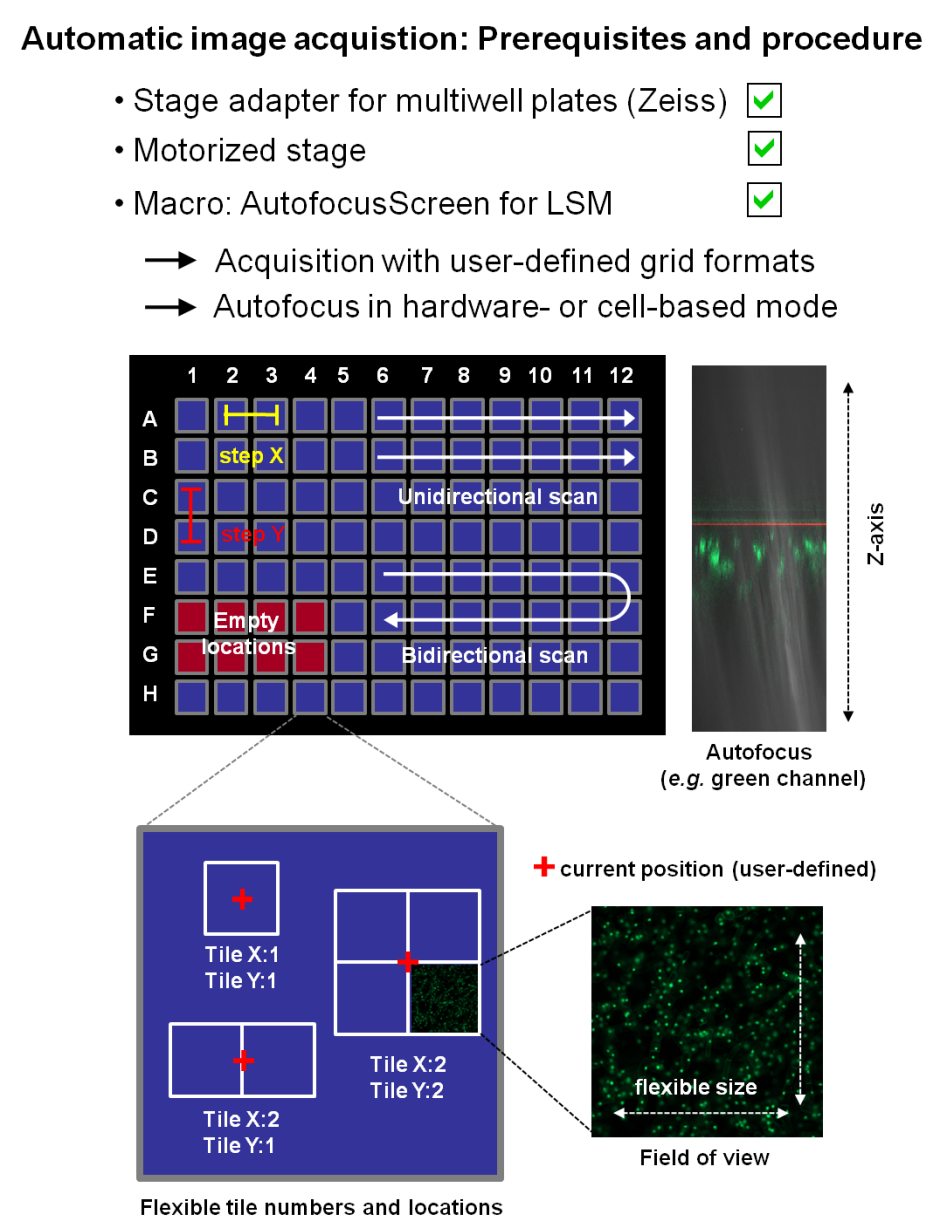
Automatic image acquisition from multiwell plates. Images from multiple locations can be taken using the “AutofocusScreen for LSM” macro, developed in collaboration between Carl Zeiss MicroImaging GMBH (Jena, Germany) and the group of Dr. Jan Ellenberg at the European Molecular Biology Laboratory (EMBL, Heidelberg, Germany). It is freely downloadable at http://www.zeiss.de/LSM-Macros. Well positions selected to be scanned can be defined by simply clicking check boxes. Steps X and Y define the distance between two acquisition locations. The exact position (here marked by a red cross) will be defined by the user, at the beginning of the image acquisition procedure. The user also has the option to define multiple tiles (with no or partial overlap to each other) around this position. The size of the scanned image will also be defined by the user and the scanning settings he chooses. Autofocus can be hardware- or cell-based, acquiring the emitted laser light or the emitted light of the sample respectively (the above shown image displays a cell-based scan across the Z-axis, using the green channel). Initial experiments with tobacco- BY-2 cells however showed that at multiple positions in the Z-axis above the glass bottom, a significant amount of cells could be visualized in the respective focal plane. This factor however will have to be adjusted for the scanning of multiple wells, as BY-2 cells will sediment quite fast (within minutes) to the bottom of the well, resulting in a significant change in the conditions and number of cells in the Z-axis.

A prerequisite for an image-based screening system is a certain degree of automatization as far as repetitive tasks are concerned. The use of the AutofocusScreen for LSM macro provided by Zeiss allows the automation of different steps of the image acquisition process. The first tests performed with the 96-well glass bottom plates indicated that all features could be used, including the autofocus routine and the automatic well-readout ( Figure 16). However, in order to find the right balance between speed and image quality, the protocol still requires refinement and further validation before reproducible and exploitable data sets may be obtained.

## Discussion

According to Carpenter ^37, 38^, in chemical screening based on fluorescence-imaging, fluorescence intensity might fluctuate from cell to cell for various reasons, including differences in cell cycle position, stochastic variations in gene expression, pre-existing amounts of proteins and metabolites in each cell and micro-environmental differences (due to cell medium or cell-to-cell-contacts). In addition, in the case of plant tissue cultures, and in particular of fast-growing cell suspension cultures, even subtle changes in the *in vitro* environment, hormonal influence or various stresses are known for their potential to influence the regulation of gene expression and even to cause fundamental alterations on the molecular level, such as chromosomal changes or DNA methylation events. Each single one of those possible factors or a combination thereof may have significant impact on the transgene expression of the individual cells within a clonally propagated suspension culture and may even result in stable genetic, epigenetic or phenotypic variation, often referred to as “somaclonal variation” ^130, 131^. The extent and the frequency of those events tend to correlate with the age of the primary callus/culture and usually increase progressively over time ^132^. However, nowadays many tools and techniques are available to detect those changes on the molecular, metabolite and phenotypic level and thus to monitor the overall fitness of the *in vitro* cultures ^133^.

In order to better understand the impact of multiple factors on the growth and expression capacity of transgenic tobacco BY-2 cells, a few of the possible causes of variations will shortly be discussed in the context of observations made during our studies.

***Expression noise and cell-to-cell variations***. Variations in the expression of proteins in a population of genetically identical cells may occur for various reasons and there are many aspects that may contribute to this behavior ^115^. For instance, several studies in bacterial and yeast model systems have shown that a certain amount of this cell-to-cell variation resulted from so-called “expression noise”, that may be defined as stochastic fluctuations in the expression of a gene ^134– 137^ focused on the expression noise in gene networks, and showed that these stochastic variations were caused by intrinsic noise at the level of the gene (e.g. number of mRNA copies), transmitted noise from upstream genes and global noise affecting all the genes.

Other studies however suggested that expression noise was rather a minor source of total cell-to-cell variations ^138^ and showed that the differences may be caused by other factors, such as the capacity of individual cells to express proteins from genes (expression capacity). For instance, differences may occur in the levels of cellular components needed for protein expression (e.g. variations in the global pool of housekeeping genes, cell cycle position or fluctuations of environmental conditions discussed later). As an example, Gordon *et al.* ^123^ monitored the expression levels and maturation rates of YFP (yellow fluorescent protein) in exponentially growing yeast cells with a pheromone-inducible gene expression system. Interestingly, they observed that the total amount of the reporter protein YFP could vary by up to a 4-fold in inducer-treated yeast cells ( *Saccharomyces cerevisiae*), whereas the maturation rates of the protein only showed little variations (39 min +/- 7 min). Even though these studies used less complex model systems than plant cells, this could explain the variations in fluorescence levels observed during this work.

***Natural heterogeneity of transgene expression in tobacco BY-2 cells***. Tobacco BY-2 cells are often referred to as the HeLa cells of plant molecular biology and were used in hundreds of studies focusing on various aspects of plant physiology ^139, 140^. Under standard growth conditions, the cell duplication time is around 14 h ^139^ and the cell divisions can be synchronized, which allows cell cycle-related studies ^140– 143^. Although they are not able to form chloroplasts and have to be grown under heterotrophic conditions, they nevertheless contain active proplastids and leucoplasts ^139^ and have been shown to be an excellent system to study the synthesis of sterols and isoprenoids ^144, 145^.

**Figure 17.**
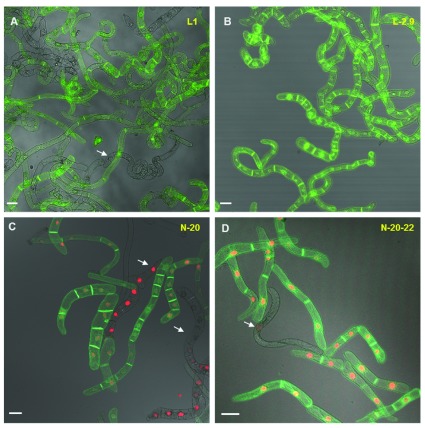
GFP and RFP fluorescence in primary and secondary suspensions. **A**- and **C**- Heterogeneity of GFP and RFP fluorescence in a suspension of transgenic BY-2 cells derived from primary calli. Arrows indicate cells with missing fluorescence or significant variations in fluorescence intensities (red and green fluorescence). **B**- and **D**- Cell suspensions derived from re-selected calli (secondary calli). The fluorescence is strong and homogenous in both channels. Nevertheless some cells (less than 5%) show heterogeneity in fluorescence. Possible reasons are discussed in the main text. White bars indicate 20 μm.

Given the higher complexity of plants, such as tobacco, compared to bacteria or yeast, variations in the expression levels of endogenous and reporter proteins from one cell to another can easily be imagined. This aspect is particularly interesting in connection with our observations, where transgene expression in individual cells of the newly generated H _6_-GFP-BD-CVIL BY-2 cell line proved to be unstable and heterogeneous in many cases ( Figure 17).

These often dramatic changes in the brightness of fluorescent cells, as well as the unstable ratio of fluorescent to non-fluorescent cells in a supposedly clonal cell line soon led us to hypothesize that the cultured cell suspensions, derived from primary calli (the first calli obtained after *A. tumefaciens*-mediated transformation), could contain (epi-)genetically different cells. The heterogeneity of callus cultures is a well-known phenomenon for many plant species and consequently, the selection of highly productive cell lines that show the desired attributes is a common step, especially for commercial efforts to produce secondary metabolites *in vitro* ^146– 150^.

Such variations in expression levels among independent transgenic lines (from the same initial transformation event) might also arise from the inserted sequence itself. Changes can indeed be induced by the methylation degree of chromosomal insertion regions ^149^, the locus of the insertion ^150^, and the number of insertion copies or transgene silencing ^151– 153^. For instance, only recently have experiments demonstrated that the integration site of a transgene significantly influences its susceptibility to RNA silencing, rather than affecting its initial expression level ^154^.

In the past, several studies ^155, 156^ helped to get a clearer picture of the variations of transgene expression in genetically identical clones. However, the most interesting contribution to this poorly understood topic came very recently from Nocarova and Fischer ^103^. They reported a method to clone transgenic tobacco BY-2 cells with the goal of reducing the high natural heterogeneity of transgene expression. The cell lines generated in their laboratory “ *repeatedly produced only a low frequency of cell lines with well-balanced and stable fluorescence in all cells*”. These observations corroborated the results obtained during the generation of both cell lines (H _6_-GFP-BD-CVIL and SV40-mRFP/H _6_-GFP-BD-CVIL) during the study presented here.

In order to identify the sources of such heterogeneity, Nocarova and Fischer ^103^ transformed tobacco BY-2 cells with a gene encoding a free GFP under the control of the CaMV 35S promoter driving the constitutive expression of the transgene. Then they monitored the expression levels of GFP in primary calli and the derived suspension cultures (primary suspensions) as well as in secondary calli and suspensions they obtained by a simple cloning/selection procedure ( Table 2). Interestingly, only about 40% of the (primary) calli obtained after *Agrobacterium*-mediated transformation showed homogenous GFP fluorescence. The remaining calli displayed heterogeneous GFP expression, either in a mosaic (m) or sectorial (s) distribution pattern within the calli. In addition, up to 90% of the (primary) suspension culture lines derived from all primary calli consisted of cells with heterogeneous levels of GFP fluorescence. On the other hand, secondary calli, obtained with their cloning method, showed homogenous fluorescence in approximately 90% of the cases, whereas only a little more than 40% of secondary suspensions had homogenous GFP fluorescence intensities.

Molecular analysis of the primary clones by Nocarova and Fischer ^103^ by Southern hybridization identified two causes for the observed heterogeneity:

The first was genetic heterogeneity due to the presence of cells with different T-DNA insertions, and the second was epigenetic heterogeneity, caused by transgene silencing at the transcriptional level in connection with DNA-methylation, as treatment with the DNA-demethylation drug 5-azacytidin ^157^ reactivated GFP expression in some lines. In many cases this heterogeneity could be resolved by subsequent cloning, but nevertheless a certain fraction showed what the authors called a “permanent expression heterogeneity” which could, for example, be due to temporal changes in the accessibility of promoter sequences to transcription factors ^151^.

To resume this part, these results are interesting in different ways: first, they might help to explain the heterogeneity in the fluorescence levels of the transgenic cell lines generated in this work. This heterogeneity was observed in our lab independently from other sources, and various approaches were discussed on how to eliminate or reduce it. Finally, we developed a simple and inexpensive method to generate secondary calli, and the resulting transgenic lines proved to be more homogenous. For the GFP-BD-CVIL line, 23 out of 48 suspension cultures (~48%) derived from primary calli showed fluorescent cells. However, the majority of these lines displayed weak ratios of fluorescent cells to non-fluorescent cells. The four lines with the best ratios and highest fluorescence intensities were chosen to reselect calli with our protocol. Two out of 15 secondary lines showed strong, homogenous fluorescence and a very high ratio of fluorescent to non-fluorescent cells ( Figure 17).

**Figure S1.**
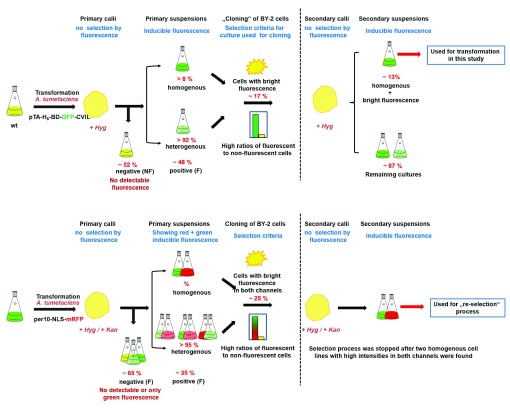
Scheme of the cloning procedure used for the selection of homogenous, transgenic tobacco BY-2 cell lines. **A** – Selection of H _6_-GFP-DB-CVIL cell line. **B** – Selection of the double-transformed H _6_-GFP-DB-CVIL/NLS-mRFP cell line. The selection of primary and secondary calli was performed on antibiotica-supplemented solid plates. As the expression of both fluorescent reporter proteins was inducible, no pre-selection by fluorescence microscopy could be used to eliminate non-fluorescent calli (it was tried by incubation of the calli in presence of the inducers, but no fluorescence could be detected with this method). The selection criteria for cultures used in the cloning procedure were based on the properties optimal for the bioassay (homogenous, bright fluorescence and high ratios of fluorescent to non-fluorescent cells.). Rounded values for statistical evaluation are indicated if available.

For the double transformation of the H _6_-GFP-BD-CVIL line with the SV40-mRFP gene construct, 20 appearing calli were screened, and seven out of them showed strong fluorescence in the first generation of cell suspension cultures (35%). In order to obtain a more homogenous line, calli from suspension cultures were reselected and four promising cell lines were obtained, but only the best-performing line was subcultured in liquid medium. In the course of such studies, the best performing lines were sub-cultured on a weekly basis as fast-growing cell-suspension cultures, as well as monthly subjected to the re-selection procedure described in Table 2 in order to obtain new batches of cell suspension cultures with homogenous, bright fluorescence. A summary of clonal selection and propagation procedures is shown in Figure S1.

***Optimization of culture conditions in microtiter plates***. Another important factor, which plays a key role in the successful set-up and miniaturization of the assay, are changes in growth conditions, such as in the inoculum ratio, agitation, aeration and temperature. Growth conditions for tobacco BY-2 cells, subcultured on a weekly basis in 250 ml Erlenmeyer flasks, were more or less constant for several years: 0.75 to 2 ml of a 7-day-old, stationary liquid suspension culture was used to inoculate 40 ml of BY-2 medium supplemented with the required selection markers and the cells were grown in the dark on a rotary shaker at 154 rpm and routinely at 26°C ^30, 145^. Nevertheless, given various problems with the culture room facilities, conditions were not always as stable as desired. In addition, downscaling the whole test system from commercial 6-well plates to high-tech glass bottom 96-well plates made it necessary to re-examine all growth conditions to determine optimal conditions for this smaller format.

For instance, ***agitation*** of plant culture cells plays an essential role as it provides homogeneity of the culture in respect to nutrients, enhances mass and heat transfer, and reduces cell clumping and formation of aggregates, a phenomenon often observed in plant cell cultures, due to secretion of extracellular polysaccharides (EPS) (see 158– 160). For example, insufficient mixing triggers the formation of aggregates and some heterogeneity in oxygen and nutrient supply inside the cell population. Therefore, sub-optimal agitation conditions may also explain the differences in transgene expression levels observed during this study.

***Aeration*** of plant suspension cultures is also an important parameter as it leads to desorption of volatile products and removes metabolic heat. Oxygen supply is hence a very crucial factor, as excess or lack can both have negative effects. Without going into detail, the mass transfer coefficient, K _La_ is a function of agitation and aeration at the same time and is part of an equation commonly used to optimize growth conditions in modern bioreactor systems ^148^. Plant cells grow relatively slowly and are known to be particularly sensitive as to their optimal oxygen supply. They adapt their metabolism even to minor changes in gas composition. This may result in an alteration of growth characteristics and in production of secondary metabolites ^146^. Under sub-optimal growth conditions, one can easily imagine that an insufficient supply of the prenylation precursor, geranylgeranyl diphosphate (GGPP) and its hydrolyzed product geranylgeraniol (GGol) in some cells could result in the shift to the nucleus of a part of the fluorescent GFP-BD-CVIL protein. This view is supported by the fact that exogenous GGol is able to completely reverse inhibition of the MEP pathway, whereas the control experiments always had a fraction (< 5%) of cells with signals from the nuclei ^161^.

Finally, the ***temperature*** is a major factor for the cultivation of plant cell cultures. Conditions are dependent on the plant species, and even for the same species, optimal temperatures may vary as far as the synthesis of a distinct metabolite is concerned ^148, 162, 163^.

Of course, all these parameters have to be adapted to the scale of the system, the cell culture line, the culture conditions and the growth phase.

***Acquisition of images at low magnification as a prerequisite***. An essential aspect and one of the novel contributions of this study was the acquisition of images at low magnification, showing groups and sub-populations of cells. The advantage of such an approach is the fact that an image can be stored and used to give the relevant biological information that might just be overseen or misinterpreted by a microscope user at a given moment. With the software ImageJ, it was possible to detect and quantify cells displaying a mislocalization of the fusion protein by a rather simple image analysis approach, using the nucleus as a reference point. But a major challenge for our approach was the detection of untreated cells, due to the fact that BY-2 cells are very diverse in shape and size and grow in files of different size. In addition, because they are growing in a liquid medium, it is nearly impossible to avoid superimposed cells, whatever the dilution. This is a hard challenge for fully automated image analysis software, even for specific plug-ins for the detection of cellular features that are adapted to simpler model systems and scenarios. Most likely a custom-made algorithm might cope with this problem and resolve it. A possible solution to the problem of cell shape heterogeneity might be the generation of protoplasts and their analysis by flux cytometry, but from our experience the necessary digestion of the cell wall would trigger stress responses critically biasing the observations.

### Limitations for inhibitor screening – the potential for identification of further molecular targets

In a previous study ^30, 31^ it had been demonstrated that inhibition of MEP pathway enzymes and of protein geranylgeranylation led to essentially identical phenomena, *viz*., the mislocalization of GFP-BD-CVIL (and of its derivative H _6_-GFP-BD-CVIL) from the plasmalemma to the nucleus. By simple chemical complementation it was possible to distinguish between inhibitors of at least the first two enzymes in the MEP pathway and isoprenylation itself. Thus, given that there is a hit in screening unknown compounds, this method could be applied again. Within a short time, the range of putative molecular targets could be minimized by such follow-up experiments. It also became obvious that the mislocalization was perfect in the presence of a low concentration of mevinolin, which suggests that the MVA pathway seems to contribute to, or promote, the formation of isoprenyl residues (< 5%). However, in an extension of the test method it is also possible to treat all cells with MEP pathway inhibitors and then screen for low molecular weight compounds that alleviate the inhibition, visualized by targeting the GFP fusion protein back into the plasmalemma, as we have seen for instance with exogenous GGol, which efficiently overcame the MEP pathway inhibition at < 5 µM. Preliminary observations argue also for a hormonal regulation of protein isoprenyl transferase activity and for some adaptation of enzymes to the pool size of substrates, indicative of some kinetic flexibility in distinguishing between C _15_ and C _20_ substrates.

The availability of this bioassay should be useful to address open questions and for many applications in agriculture and medicine. For instance, one can imagine applying this test to the search of compounds that interfere with the bio-activation of the herbicide clomazone to oxoclomazone, which is P450-mediated, or more generally the search of new herbicides. To elucidate mechanisms underlying the intracellular transport of prenylated protein in BY-2 cells is also an interesting topic. According to Gerber ^161^, the classical secretory pathway appeared not to be involved as a transit route of the GFP-BD-CVIL to the plasmalemma of BY-2 cells. Interestingly, human KRAS4B shares several features with the GFP-BD-CVIL fusion protein ( *e.g.* the polybasic domain and the CaaX-motif) ^164, 165^. Several prenyl-binding proteins are known, such as Rho-GDI or the δ-subunit of phosphodiesterase (PDEδ) in animals ^166– 168^ that are involved in the binding of isoprenylated Ras protein and the transport to its final membrane destination. Similar proteins might be candidates for the transport of H _6_-GFP-BD-CVIL from the ER to its final cellular destination. The use of our bioassay in combination with inhibitors of CaaX-processing and compounds interfering with the membrane anchorage of H _6_-GFP-BD-CVIL might be a promising strategy to investigate this aspect in the development of anti-cancer strategies.

### Further usefulness of the BY-2 cell-based visualization method

**Figure 18.**
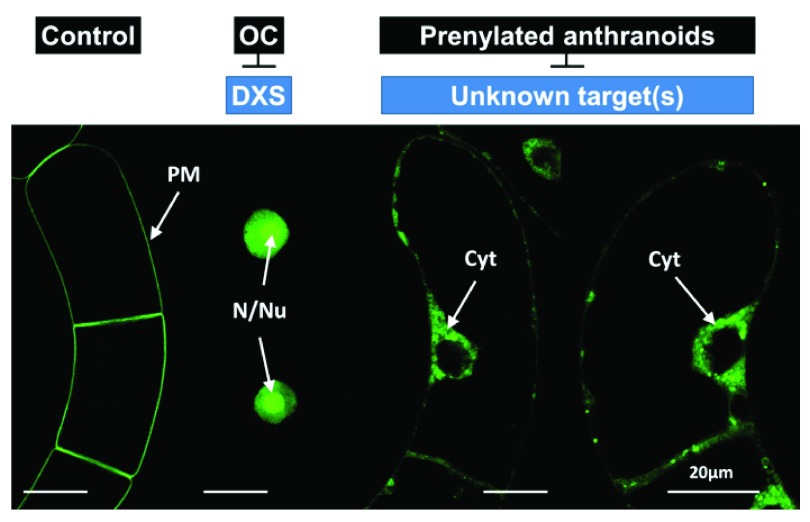
Confocal images indicating the unusual distribution of H _6_-GFP-BD-CVIL in response to a prenylated anthracenoid derived from an African medicinal plant. On the left side as controls cells without and with oxoclomazone (OC) treatment showing plasma membrane (PM) localization or its mislocalization to the nucleus (N), especially in nucleoli (Nu). On the right side cells are depicted that have been treated with an inhibitor that has shown a high efficiency in killing protozoan *Leishmania* cells, responsible of leishmaniasis. The H _6_-GFP-BD-CVIL is mainly accumulated in the cytoplasm (Cyt) that is for instance surrounding the nucleus. At the higher magnification some punctuate structures become visible. That might result from coagulated cytoplasm typical of cell death. White bars in the combined images represent 20 µm.

The bioassay may also be very useful to screen unknown chemical compounds with cytotoxic properties. By testing potential inhibitors derived from plant extracts of the *Clusiaceae* family that are traditionally used for the treatment of parasitic diseases in Cameroon, we made an interesting observation ( Figure 18). Whereas none of the compounds was efficiently inducing a mislocalization of the fusion protein to the nucleus (compare OC treatment), several treatments triggered toxic effects. One compound in particular displayed an unusual phenotype, with barely detectable signals from the plasma membrane (PM) and nucleus (N/Nu); instead, the GFP fluorescence seemed to be trapped in the cytoplasm (Cyt). Interestingly, this compound was identified as a prenylated anthracenoid, isolated from *Psorospermum glaberrium* (Clusiaceae) found in Cameroon, and shown to have the highest activity in tests against leishmaniasis ^169^, further validating the experimental system as a tool to detect cytotoxic effects. This could be an indication that the transport of H _6_-GFP-BD-CVIL might be mediated by protein-protein interactions, which are impaired by the prenylated compound.

Anthroquinolol derivatives being structurally similar to the above-mentioned anthracenoid, isolated from *Antrodia camphorata*, an endemic fungus found in Taiwan, have very recently been shown to block Ras and Ras-related GTP-binding protein activation in human lung cancer (A549 and H838), liver cancer (HepG2 and Hep3B), and leukemia (K562 and THP-1) cell lines by direct binding to FTase and to GGTase I ^170^.

***Comparison with other screening methods for inhibitors of the MEP pathway and potential for further applications***. Screening methods for MEP pathway inhibitors have already been described in the past, but were based on *in vitro* assays, using enzymes from *E. coli* alone and fluorescent compounds ^171^ or in combination with downstream enzymes of the MEP pathway together with auxiliary enzymes necessary for optical measurements in medium-throughput methods. Extracts from a series of Mediterranean plants were for instance examined for their inhibitory potency of *E. coli* MEP synthase (ispC = deoxyxylulose phosphate reductoisomerase, DXR), with the most efficient one coming from *Cercis siliquastrum* ^172^. However, the truly active individual components could not yet be identified. A similar system based on cloned and heterologously expressed and combined DXS and DXR from *Mycobacterium tuberculosis* was recently described ^173^. While such approaches are certainly suited to identify inhibitors that react with an enzyme, their activity *in vivo* could well be disappointing if they are not absorbed to penetrate internal cell membranes to arrive at their molecular target(s). This is the principal advantage of using a whole-cell method, as described in this article. We have recently become aware of a faintly related approach, based on the measurable growth inhibition of specifically engineered strains of *Salmonella typhimurium* having a separately inducible (native) MEP pathway and an inducible (non-native) MVA-utilizing pathway ^174^. However, compounds that are efficient in a plant cell-based system like oxoclomazone ^30^ are only mildly or not at all inhibitory to bacterial counterparts of MEP pathway enzymes. Furthermore, our screening system is also suitable to check for inhibitors of protein geranylgeranylation, an important aspect in for instance cancer research ^175^ and even in studies on the recruitment of the eukaryotic cell protein isoprenylation machinery by pathogenic bacteria injecting isoprenylatable proteins (cf. 7, 8, 21). More recently, a test system was described, based on inhibitors affecting the accumulation of phytoene in barley leaf cuttings, after treatment of plants with a known herbicide (norflurazon at 200 µM) that block the conversion of this intermediate into end-of-chain carotenoids ^176^. By this approach compounds that interfere with the accumulation of phytoene at steps preceding phytoene synthase could be detected, which was verified by applying representative inhibitors like derivatives of clomazone. However, the procedure required the rather tedious extraction and separation from lipids by saponification, another solvent extraction from the MeOH/KOH solution and finally the photometric quantification ^176^.

### Materials and methods

The sources of chemicals and biochemicals were already described in the preceding article ^32^, as well as the basic methods like cultivation of tobacco BY 2 cells, their stable transformation by a gene encoding H _6_-BD-GFP-CVIL placed under the control by a dexamethazone-inducible promoter. This also holds true for microscopic techniques. Only what methods had been newly introduced in this study will be described here.

***DXR and HMGR artificial microRNA silencing strategy***. Artificial microRNAs (amiRNA) designed to silence DXR and HMGR genes were predicted using the Web MicroRNA designer WMD2. For DXR, we used the *Nicotiana tabacum* DXR cDNA sequence (Genbank accession number DQ839130) as a template and four potential miRNA were selected. For the design of amiRNA capable of silencing all of the HMG-CoA reductase (HMGR) genes *HMGR1-* NTU60452, *HMGR2*- AF004232, *HMGRL*- AF004233, looked for the regions with highest homology between all of the isoforms and submitted it to WMD2. In this case, two potential miRNAs were selected. The selected ami-RNA sequences were amplified by recursive PCR as described by Schwab *et al.* ^118^ using the primers depicted in Table 3 and Table 4, and cloned into pBSK.

**Table 3.** Sequences for DXR amiRNAs.

Name	Sequence	Bp
DXR 1.I	gaTTACTTTACATTATCAGGCGCtctctcttttgtattcc	40
DXR 1.II	gaGCGCCTGATAATGTAAAGTAAtcaaagagaatcaatga	40
DXR 1.III	gaGCACCTGATAATGAAAAGTATtcacaggtcgtgatatg	40
DXR 1.IV	gaATACTTTTCATTATCAGGTGCtctacatatatattcct	40

DXR 3.I	gaTTATATCCAACGTCTGAGCTCtctctcttttgtattcc	40
DXR 3.II	gaGAGCTCAGACGTTGGATATAAtcaaagagaatcaatga	40
DXR 3.III	gaGAACTCAGACGTTCGATATATtcacaggtcgtgatatg	40
DXR 3.IV	gaATATATCGAACGTCTGAGTTCtctacatatatattcct	40

DXR 4.I	gaTAACAGTAAATTCTGCCGGCCtctctcttttgtattcc	40
DXR 4.II	gaGGCCGGCAGAATTTACTGTTAtcaaagagaatcaatga	40
DXR 4.III	gaGGACGGCAGAATTAACTGTTTtcacaggtcgtgatatg	40
DXR 4.IV	gaAAACAGTTAATTCTGCCGTCCtctacatatatattcct	40

DXR 7.I	gaTCATTTACTACGATAGACGTTtctctcttttgtattcc	40
DXR 7.II	gaAACGTCTATCGTAGTAAATGAtcaaagagaatcaatga	40
DXR 7.III	gaAAAGTCTATCGTACTAAATGTtcacaggtcgtgatatg	40
DXR 7.IV	gaACATTTAGTACGATAGACTTTtctacatatatattcct	40

**Table 4.** Sequences for HMGR amiRNAs.

Name	Sequence	Bp
HMGR 3.I	gaTTTACGAATAGAAGGAGCCCTtctctcttttgtattcc	40
HMGR 3.II	gaAGGGCTCCTTCTATTCGTAAAtcaaagagaatcaatga	40
HMGR 3.III	gaAGAGCTCCTTCTAATCGTAATtcacaggtcgtgatatg	40
HMGR 3.IV	gaATTACGATTAGAAGGAGCTCTtctacatatatattcct	40

HMGR 5.I	gaTACGAATAGAAGCAGCCCCCTtctctcttttgtattcc	40
HMGR 5.II	gaAGGGGGCTGCTTCTATTCGTAtcaaagagaatcaatga	40
HMGR 5.III	gaAGAGGGCTGCTTCAATTCGTTtcacaggtcgtgatatg	40
HMGR 5.IV	gaAACGAATTGAAGCAGCCCTCTtctacatatatattcct	40

The amiRNA sequences were subcloned into the pER10 inducible vector ^86^, kindly provided by Prof. Nam-Hai Chua, Rockefeller University) by XhoI-SpeI and transformed into an *Agrobacterium tumefaciens* LBA4404 hypervirulent strain. *Agrobacterium* transformants were checked by PCR (using oligos pER for 5’- GCTCGACTCTAGGATCTTCG - 3’and pER rev 5’- GTAGGATTCTGGTGTGTGG-3’) and used for stable transformation of BY-2 cell lines.

Stable BY-2 cell lines expressing the construct H _6_-GFP-BD-CVIL were previously reselected for good fluorescent intensity. 3-day-old selected cell suspensions were transformed through co-culture with pER10-DXR or pER10-HMGR transformed agrobacteria and plated into MS-hygromycin/kanamycin (30 µg/ml each). Plates were incubated in the dark at 28°C until calli appeared. At least 10 transformed calli were re-selected three times through fresh MS plates (supplemented with hygromycin and kanamycin, both at 30 µg/ml) and derived minicultures were sub-cultured weekly (10 ml, MS supplemented with hygromycin and kanamycin, both at 30 µg/ml). Genomic DNA was extracted from each BY-2 clone and t-DNA insertion was checked by PCR (using oligos pER for and pER rev). For fluorescence screening we first treated the cells with 5 µM estradiol (Est) (to induce amiRNA expression) and/or 50 µM fosmidomycin (FOS), as control of the expected phenotype. After 2 h, GFP fluorescence was induced by addition of Dex at 10 µM final concentration and cell cultures were screened by confocal laser scanning microscopy for lines showing high levels of GFP fluorescence and also exhibiting a DXR silencing phenotype (e.g. mislocalization of the H _6_-GFP-BD-CVIL from the plasma membrane to the nucleus). HMGR silenced lines were selected among those lines showing high levels of GFP fluorescence at the plasma membrane. Selected lines were subsequently used for inhibition tests, including treatments with EST, FOS or mevinolin (MEV).

As a control of such silencing experiments, stably transformed lines were generated with the pER10 empty vector. Induction by EST did not result in mislocalization of H _6_-GFP-BD-CVIL from the plasma membrane to nuclei, as expected.

Gene expression was analyzed by quantitative reverse transcriptase-mediated Real Time PCR (qRT-PCR). RNA was extracted from dried BY-2 cells with the Nucleospin RNA Plant kit (Macherey-Nagel) and cDNAs were synthesized from 1 μg total RNA by using a SuperScript III Retro transcription kit (Invitrogen). *DXR* and *HMGR* gene expression was analyzed by qRT-PCR, using SYBR green (MESA GREEN + fluorescein, Eurogentec) in the iCycler thermal cycler (Biorad). *EF1* and *actin* were used as control genes. Primers used for qPCR analysis were designed with Primer3 public software ( http://frodo.wi.mit.edu/primer3/). Primer sequences for *HMRG, EF1* and *actin* genes were described ^119^ and *DXR* primer sequences are as follows: DXR-F 5’-GCTGAGAATCCGGACAAGTT-3’ and DXR-R 5’ TTTTGGTTTGAATGTTTTGACC-3’. Experiments were repeated at least three times. RNA quality was monitored by UV spectrometry.
