# Supplementary material for: Development of an image-based screening system for inhibitors of the plastidial MEP pathway and of protein geranylgeranylation
Source: F1000Res. 2015 Aug 12;4:14. Originally published 2015 Jan 16. [Version 2] doi: 10.12688/f1000research.5923.2 (PMC4536634; doi:10.12688/f1000research.5923.2)
Supplement: Peer review history

# Peer review: Referee response for version 1

**DOI:** 10.5256/f1000research.6329.r8261

**Published:** 2015-04-16

**Authors:** Wolfgang Eisenreich

**Affiliations:** 1 Department of Chemistry, Technische Universität München, München, Germany

The article provides detailed information about an *in vivo *based assay system of protein prenylation using stably transformed tobacco BY-2 cells. Notably, the authors show that the method is also useful in finding potential inhibitors against enzymes catalyzing upstream reactions in the MEP pathway of IPP/DMAPP biosynthesis. On this basis, the assay is a welcome addition to existing high-throughput screening assays using photometry. The paper is well written and nicely illustrated. I congratulate the authors on their findings.

I have read this submission. I believe that I have an appropriate level of expertise to confirm that it is of an acceptable scientific standard.

# Peer review: Referee response for version 1

**DOI:** 10.5256/f1000research.6329.r7343

**Published:** 2015-03-19

**Authors:** Patricia León

**Affiliations:** 1 Departamento de Biología Molecular, Instituto de Biotecnología, Universidad Nacional Autónoma de México, Morelos, Mexico

The manuscript by Hartmann *et al*. describes the establishment of an *in vivo* assay using plant cell culture cell system to estimate the geranylgeranylation of proteins. The geranylgeranilation is a posttranslational modification required for particular targeting and interactions of some proteins but in plants its function is not fully characterized. Interestingly, previous work by the same group has demonstrated that this modification relays in the activity of the chloroplast isoprenoid MEP pathway and not in the Mevalonate pathway, that operates in the cytoplasm. This manuscript describes the implementation of an *in vivo* method that allows screening for novel drugs or external signals that affect the isoprenylation process. This method takes advantage of the change in the subcellular localization of a genarygeranylated protein from the cytoplasm to the nucleus. The authors analyze different parameters in order that this bioassay could be use in a qualitative and quantitative manner. In particular this is interesting because there are not too many examples that uses plant culture cells for massive screenings based on fluorescence.

My impression is that this manuscript reports a valuable methodology that will be of interest for many groups interested in understanding basic aspects of the prenylation and isoprenoid synthesis in general, and also for selection of novel inhibitors of the pathway. The data included is of good quality data and also this manuscript provides a very detailed discussion of the different parameter of the implemented system. However, a general comment of this manuscript is that is very long for the reader. I consider that some aspects could be shortened without loosing critical points.  For example, the general considerations from the introduction (page 3) could be shortened. This is also true for some points in the Results and Methodology such as the description of the ImageJ method or the modified RFP reporter protein that perhaps does not require to be described in such detail and just providing the corresponding references or refer to the related table might be sufficient.

- Another aspect I found unusual from the manuscript is that in the Discussion the authors again included additional results. I think that the authors should present the Results in the corresponding section. For example the influence of the putative methylation over the cells is an aspect that I think should be described in the Result section.  

Additionally of these general aspects related to the organization of the manuscript I have some major points that would be important to address:

- The authors select a secondary calli line that is homogenous in the fluorescence and nicely demonstrate that this line exhibits high fluorescence ratio. An important aspect to described is how stable this secondary suspension culture is and how long have the authors followed it. I wonder whether this could be a problem in the transgene expression over time. It is well know that liquid cultures tend to have polyploidization, chromosomal aberrations and maybe changes in chromatin condition. I think this is an important information to be included, particularly if somebody is interested in the selected line. The authors mention some of this aspect in the Discussion but its stability over time is an important information.

- To reinforce that the changes in localization observed with the inhibitors is due to a specific blockage of the MEP pathway the authors also used microRNA interference of the *HMGR* and *DXR* genes. The authors report that the localization of the GFP-BD_CVIL is very similar to that observed with fosmidomycin and oxoclomasone, supporting the role of the MEP pathway in the geranylgenarlynation. However in the case of the ami HMGR the authors state to not detect any change in the localization. If I understand correct no difference in localization was found with the MVA inhibitor (Figure 9G) and this is exactly what they were expecting. From this result the authors discuss about the inhibition levels for the HMGR that appear to be limited and the possible role of sterols for silencing. I think that this aspect in the current form and probably requires rewriting. Also since the levels of RNA the different ami lines tested are not shown this part remains not fully clear. Thus I think that if this aspect remains the authors should at least mention the inhibition level observed in each line.

Minor comment

- In the abstract the authors state “Furthermore, complementation assays with pathway-specific intermediates confirmed that the precursors for the cytosolic isoprenylation of this fusion protein are predominantly provided by the MEP pathway” Maybe it escapes me but I did not find any mention in the text of complementation with the intermediates and maybe this paragraph maybe deleted from the Abstract.
- Page 10. As a negative control the cells were treated with the plasma membrane stain FM4-64. I presume that this data is not shown and this should be indicated in the text.
- Page 10. In the induction with 6µM Est the time laps used maybe better to be reported in increasing order after induction.
- In the legend of Figure 7 it is stated “Saturation is already reach after 24 h of induction with both elicitors”. Since both elicitors are not shown in this Figure as stated before I suggest that this statement is moved to the text and not in the Legend.

I have read this submission. I believe that I have an appropriate level of expertise to confirm that it is of an acceptable scientific standard.

# Response

**Published:** 2015-07-23

**Authors:** Thomas J. Bach

**Affiliations:** IBMP, CNRS UPR 2357, Université de Strasbourg, France

First of all we would like to thank Prof. León for her thoughtful comments and suggestions to further improve the manuscript. We are aware that the manuscript is very detailed in some sections. In fact we faced some dilemma, namely to make specialists in the field of plant isoprenoid biosynthesis and function acquainted with rather advanced imaging techniques (and in this special case even with aspects of protein isoprenylation in general), which might not be in the focus of phytochemists and plant physiologists. At the same, specialists in imaging techniques might also be interested in the particular situation of subcellular metabolic organization in plant cells and the challenges arising from the use of living plant cell cultures that had to be adapted to such analyses, for instance through diminishing the size of cultures to fit into 96-well plates. We would not expect the gentle readers to analyze the publication systematically, but rather to focus on those aspects that seem particularly interesting or where detailed information is essential for evaluation and understanding.

As to the DNA methylation problem: We will reorganize the manuscript accordingly. This data was originally integrated in a supplementary part and only later found its way into the main manuscript, which led now to this justified criticism by Prof. León. This part has now been integrated into the Results section where we think it fits.

As to selection of call and so forth:  Admittedly, this aspect cannot be neglected and additional information concerning the process of generation and maintenance of the cell cultures will be included in the updated version of the manuscript, in particular a scheme illustrating the process of obtaining the doubly transformed cell lines used in this study (Supplemental Figure 1). 

As far as the maintenance of the cell lines is concerned, we needed to restart liquid cultures from calli several times to recover good levels of fluorescence. This correlated well with documented failures in the maintenance of temperature in the growth chambers. As mentioned in the manuscript, the main culprits for the observed loss of fluorescence were likely changes on the molecular level, in particular DNA methylation events, which became evident after the prolonged treatment with 5-azacytidine recovered some red fluorescence. However, at the same time it led to cell death in a considerable number of cells.

We cannot exclude that other, more severe events such as chromosomal alterations might have contributed as well to the loss in fluorescence. We were aware of this possibility but decided to focus on a quick way to save and re-select our “precious” cell lines from cells that might not have been affected by those “somaclonal” events instead of using the whole repertoire of available techniques to figure out the individual causes for those changes.

In addition, it should not be forgotten that maintaining the cell culture as a suspension in the long term is time-consuming and costly and does not assure high and homogenous levels of transgene expression. Thus, cell suspension establishment from (re-)selected calli - as suggested in the manuscript - was implemented as a standard procedure once the protocol for it was fully optimized and is highly recommendable. 

As for the amiRNA constructs now expression data has been added. Up to 10 lines per construct were selected and tested and none achieved better levels of silencing than those presented. It should be noted however that this was not the main point goal of the publication! 

If we take for instance these amiRNA experiments, then the initial idea was based on a request by a reviewer commenting on the publication that appeared in 2009 (Gerber et al. – reference 30 in the article). To us it was more important to show an independent activation of promoters by dexamethasone (GFP-CVIL/M) and the amiRNA construct to silence either HMGR or DXR (cf. Figure  11 new). The next step was then to express a nucleus-targeted RFP under the control of estrogen, without any cross induction…

We discussed this part in view of more recent observations that suggested some dependence of the RNAi machinery on the presence of sterols in the membrane to which essential proteins in this process. One Arabidopsis mutant was deficient in HMGR, known to coarse-regulate sterol biosynthesis. Thus the inhibition by mevinolin might have mimicked such an effect, and thereby a “negative“ result (means no visible inhibition by amiRNA) would be no proof per se. We think that this aspect is properly discussed, even though the visible outcome is what was more or less “expected“ (see Gerber et al. 2009). We admit that the percentage of inhibition (quantitation of corresponding mRNA by PCR) was limited, which in the case of HMGR silencing could also be interpreted to mean that inhibition would be partial at best. By the way, when such an approach had been used with an enzyme downstream of HMGR, *viz*., cycloartenol synthase (CAS) it resulted in about 35% diminished mRNA, that of HMGR1 and 2 by about 60% after induction by estradiol ( Gas-Pascual *et al*., 2015). This paper is now quoted accordingly.

The criticized part in the Abstract relates to the preceding publication, in which such inhibitors had been specifically tested, also chemical complementation by addition of intermediates whose formation had been blocked. At the same this approach could be extended in adding effector molecules, i.e. hormones and growth regulators and watch of whether the mislocalization of the indicator protein could be overcome, for instance through activation of pathways and transporters…  Addition of “preceding” where needed should now have clarified why this part was added to the Abstract. A citation number was not allowed to be added by the publisher.

The staining by FM4-64  is shown in figure 6, and this information has been added once more where appropriate.

The time laps problem: A misunderstanding? No, We respectfully disagree - because the values are related to the time point when the cells were observed after treatment, thus this sequence is more logical as it describes the real time course of this experiment.

As to Figure 7: We feel the statement in the legend is sufficiently clear: Both elicitors were used, however the images were only examined for the red fluorescence induced by estradiol.
